# Estrogen Receptor Status Oppositely Modifies Breast Cancer Prognosis in *BRCA1/BRCA2* Mutation Carriers Versus Non-Carriers

**DOI:** 10.3390/cancers11060738

**Published:** 2019-05-28

**Authors:** Michal Vocka, Martina Zimovjanova, Zuzana Bielcikova, Petra Tesarova, Lubos Petruzelka, Martin Mateju, Ludmila Krizova, Jaroslav Kotlas, Jana Soukupova, Marketa Janatova, Petra Zemankova, Petra Kleiblova, Jan Novotny, Bohuslav Konopasek, Martina Chodacka, Milan Brychta, Marek Sochor, Denisa Smejkalova-Musilova, Vlastimila Cmejlova, Renata Kozevnikovova, Lenka Miskarova, Sona Argalacsova, Lenka Stolarova, Klara Lhotova, Marianna Borecka, Zdenek Kleibl

**Affiliations:** 1 Department of Oncology, First Faculty of Medicine, Charles University and General University Hospital, U Nemocnice 499/2, 128 00 Prague 2, Czech Republic; martina.zimovjanova@vfn.cz (M.Z.); zuzana.bielcikova@vfn.cz (Z.B.); petra.tesarova@vfn.cz (P.T.); lubos.petruzelka@vfn.cz (L.P.); martin.mateju@vfn.cz (M.M.); ludmila.krizova@vfn.cz (L.K.); bohuslav.konopasek@vfn.cz (B.K.); 2 Institute of Biology and Medical Genetics, First Faculty of Medicine, Charles University and General University Hospital, Albertov 4, 128 00 Prague 2, Czech Republic; jaroslav.kotlas@vfn.cz (J.K.); pekleje@lf1.cuni.cz (P.K.); 3 Institute of Biochemistry and Experimental Oncology, First Faculty of Medicine, Charles University, U Nemocnice 5, 128 00 Prague 2, Czech Republic; jana.soukupova@lf1.cuni.cz (J.S.); marketa.janatova@lf1.cuni.cz (M.J.); petra.zemankova@lf1.cuni.cz (P.Z.); lenka.stolarova@lf1.cuni.cz (L.S.); klara.lhotova@lf1.cuni.cz (K.L.); marianna.borecka@lf1.cuni.cz (M.B.); 4 Department of Surgery, Sunderby Hospital, Sjukhusvägen 10, 954 42 Sunderbyn, Sweden; onkologie@seznam.cz; 5 Department of Oncology, Chomutov Hospital, Kochova 1185, 430 01 Chomutov, Czech Republic; martina.chodacka@kzcr.eu; 6 Department of Radiotherapy and Oncology, Third Faculty of Medicine, Charles University and Faculty Hospital Kralovske Vinohrady, Srobarova 1150/50, 100 34 Prague 10, Czech Republic; brychta@fnkv.cz; 7 Department of Oncology, Vzdusna 1360/6, 460 01 Liberec, Czech Republic; sochor.marek73@gmail.com; 8 Department of Oncology, Masaryk Hospital, Socialni pece 3316/12, 401 13 Usti nad Labem, Czech Republic; denisa.smejkalovamusilova@kzcr.eu; 9 Department of Oncology, Second Faculty of Medicine, Charles University and Motol University Hospital, V Uvalu 84, 150 06 Prague 5, Czech Republic; v.cmejlova@seznam.cz; 10 Department of Oncology, Medicon, Roskotova 1717/2, 140 00 Prague 4, Czech Republic; r.kozevnikovova@email.cz; 11 Department of Oncology, First Faculty of Medicine, Charles University and Thomayer Hospital, Videnska 800, 140 59 Prague 4, Czech Republic; lenka.mockova@gmail.com; 12 Institute of Radiation Oncology, First Faculty of Medicine, Charles University and Hospital Na Bulovce, Budinova 2, 180 00 Prague 8, Czech Republic; argi@centrum.cz

**Keywords:** breast cancer, BRCA1, BRCA2, germline mutations, estrogen receptor, survival

## Abstract

Breast cancer (BC) prognosis in BRCA1 and BRCA2 mutation carriers has been reported contradictorily, and the significance of variables influencing prognosis in sporadic BC is not established in BC patients with hereditary BRCA1/BRCA2 mutations. In this retrospective cohort study, we analyzed the effect of clinicopathological characteristics on BC prognosis (disease-free survival [DFS] and disease-specific survival [DSS]) in hereditary BRCA1/BRCA2 mutation carriers. We enrolled 234 BRCA1/BRCA2 mutation carriers and 899 non-carriers, of whom 191 carriers and 680 non-carriers, with complete data, were available for survival analyses. We found that patients with ER-positive tumors developed disease recurrence 2.3-times more likely when they carried a BRCA1/BRCA2 mutation (23/60; 38.3% ER-positive carriers vs. 74/445; 16.6% ER-positive non-carriers; *p* < 0.001). ER-positive mutation carriers also had a 3.4-times higher risk of death due to BC compared with ER-positive non-carriers (13/60; 21.7% vs. 28/445; 6.3%; *p* < 0.001). Moreover, prognosis in ER-negative BRCA1/BRCA2 mutation carriers was comparable with that in ER-positive non-carriers. Our study demonstrates that ER-positivity worsens BC prognosis in BRCA1/BRCA2 mutation carriers, while prognosis for carriers with ER-negative tumors (including early-onset) is significantly better and comparable with that in ER-positive, older BC non-carriers. These observations indicate that BRCA1/BRCA2 mutation carriers with ER-positive BC represent high-risk patients.

## 1. Introduction

Approximately 5–10% of all breast cancers (BC) have a hereditary background [1,2]. Mutations in *BRCA1* or *BRCA2* (*BRCA1/BRCA2*) account for most hereditary BC cases [3]. The proportion of *BRCA1* vs. *BRCA2* mutations is population-specific, with *BRCA1* mutations being dominant among Czech patients [4]. Women carrying *BRCA1/BRCA2* mutations have a 70–80% risk of BC development by age 80 [5,6]. Besides a high lifetime BC risk, mutation carriers are threatened by early BC onset and an increased risk of other cancers, including ovarian cancer (OC) [7].

Breast tumors are classified into distinct molecular subtypes with different prognoses and require specific therapeutic approaches [8,9]. Most *BRCA1*-associated BC cases have typical histopathological features including high-grade and triple-negative tumors [10,11,12,13]. Triple-negative breast cancer (TNBC) accounts for 15–20% of all BC cases and is associated with worse overall survival (OS) [14,15]. Pathological characteristics of BC in *BRCA2* mutation carriers are less indicative (higher tumor grade, frequent ER-positivity, and HER-2 negativity), resembling sporadic tumors [11,12,13,16].

BC prognosis in *BRCA1/BRCA2* mutation carriers has been contradictorily reported to be worse [13,17,18] or the same [19,20,21] as in patients with sporadic disease. Recent meta-analysis comparing survival in BC patients with *BRCA1/BRCA2* mutations and non-carriers or unselected BC patients revealed that current evidence does not suggest worsened BC survival in mutation carriers [17]. The only prospective POSH (Prospective Outcomes in Sporadic versus Hereditary breast cancer) study of 2733 young-onset BC patients found no difference in OS in 338 *BRCA1/BRCA2* mutation carriers [19]. Indeed, among 558 TNBC patients, *BRCA1/BRCA2* mutation carriers had better OS than non-carriers at two years (95% vs. 91%; HR 0.59; 95% CI 0.35–0.99), but comparable at subsequent time points.

While germline *BRCA1/BRCA2* mutations undoubtedly increase BC risk, it is unclear whether the overall prognosis and clinicopathological prognostic factors differ between mutation carriers and non-carriers. This question is of considerable clinical importance because the age at BC onset is over a decade lower among mutation carriers than in non-carriers [11,12,22].

To determine clinicopathological characteristics influencing BC prognosis (disease-free survival [DFS] and disease-specific survival [DSS]), we analyzed 1133 Czech BC patients, including 234 *BRCA1/BRCA2* mutation carriers and 899 non-carriers.

## 2. Results

### 2.1. Patient and Tumor Characteristics

Among 1133 enrolled BC patients, 234 (19.5%) were carriers of pathogenic *BRCA1* (*N* = 183) or *BRCA2* (*N* = 51) mutations (Supplementary Table S1). The remaining 899 BC patients (74.8%) were non-carriers of mutations in the established cancer-susceptibility genes (incl. *BRCA1*, *BRCA2*, *PALB2*, *CHEK2*, *ATM*, *TP53*, *RAD51C*, *RAD51D*, *BRIP1*, *MLH1*, *MLH3*, *NBN*, *NF1*). Median follow-up of 1133 patients eligible for subsequent analyses was 9.8 years. A comparison of the clinicopathological characteristics of *BRCA1/BRCA2* mutation carriers and non-carriers is summarized in Supplementary Table S2. *BRCA1/BRCA2* mutation carriers were diagnosed with BC at an earlier age, with more advanced disease, different BC morphology, higher grade, and more frequent TNBC. These differences were driven mainly by *BRCA1* mutation carriers, as *BRCA2* mutation carriers differed from non-carriers only in terms of higher tumor stage and lower HER2-positivity. *BRCA1/BRCA2* mutation carriers were more often treated by chemotherapy; however, surgical treatment and radiotherapy were comparable in both groups. *BRCA1/BRCA2* mutation carriers also developed distant metastases and second BC more often than non-carriers, but the frequency of loco-regional recurrences was similar. Differences in age at diagnosis between mutation carriers and non-carriers are displayed in Supplementary Figure S1.

### 2.2. Prognosis and Long-Term Survival

**Figure 1:**
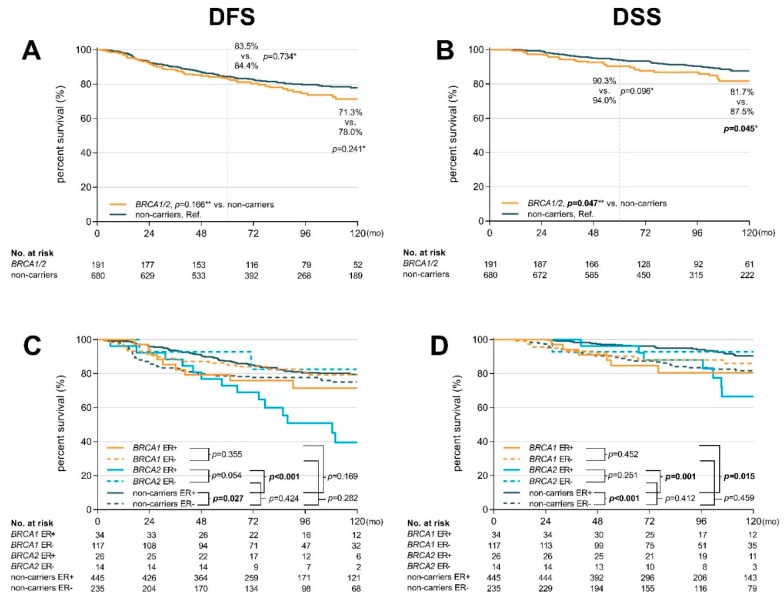
Kaplan-Meier plots showing disease-free survival (DFS) (**A**) and disease-specific survival (DSS) (**B**) in *BRCA1/2* mutation carriers and non-carriers; DFS (**C**) DSS (**D**) of *BRCA1* mutation carriers, *BRCA2* mutation carriers, and non-carriers classified according to the ER status. * *p*-values calculated by χ^2^ test (number of events at the end of follow-up interval); ** *p*-values calculated by log-rank test (considering whole follow-up period).

Complete clinicopathological data for survival analyses have been eligible for 191 mutation carriers (151 *BRCA1* and 40 *BRCA2* mutation carriers) and 680 non-carriers (Table 1) with median follow-up of 8.3 years. *BRCA1/BRCA2* mutation carriers had marginally worsened DSS (HR 1.65 95% CI 1.01–2.70; *p* = 0.047; Figure 1B) with an absolute difference of 81.7% versus 87.5% (in non-carriers) after 10 years (*p* = 0.045), already apparent at 5 years. The non-significant difference in DFS (Figure 1A) reached absolute values of 71.3% versus 78.0% (*p* = 0.241) for *BRCA1/BRCA2* mutation carriers and non-carriers, respectively. DFS was affected mainly by a higher relapse rate in carriers of mutations in *BRCA2* (but not in *BRCA1*; Supplementary Figure S2).

Next, we compared the effect of clinicopathological characteristics on survival between *BRCA1/BRCA2* mutation carriers and non-carriers by the Mantel–Haenszel test (Table 2). Older *BRCA1/BRCA2* mutation carriers had increased risk of disease recurrence (DFS), including patients ≥35 years (HR 1.81 95% CI 1.10–2.99), patients ≥45 years (HR 3.98 95% CI 1.62–9.81), and postmenopausal patients (HR 3.72 95% CI 1.16–11.90), when compared with age-matched non-carriers. Mutation carriers with ER-positive tumors also had significantly worse DFS (HR 3.14 95% CI 1.69–5.81; *p* = 0.003) than non-carriers with ER-positive tumors, with similar significant differences also detected for DSS (HR 5.70 95% CI 2.27–14.4; *p* < 0.001). The opposite prognostic effect in *BRCA1/BRCA2* mutation carriers and non-carriers was not limited to ER status only as we have observed the same trend with age at diagnosis and menopausal status. Subsequent analyses within each group confirmed the observed differences in DFS with similar trends for DSS (Supplementary Table S3). These univariate analyses also confirmed the expected negative impact of increased tumor stage on survival in both analyzed groups.

**Figure 2:**
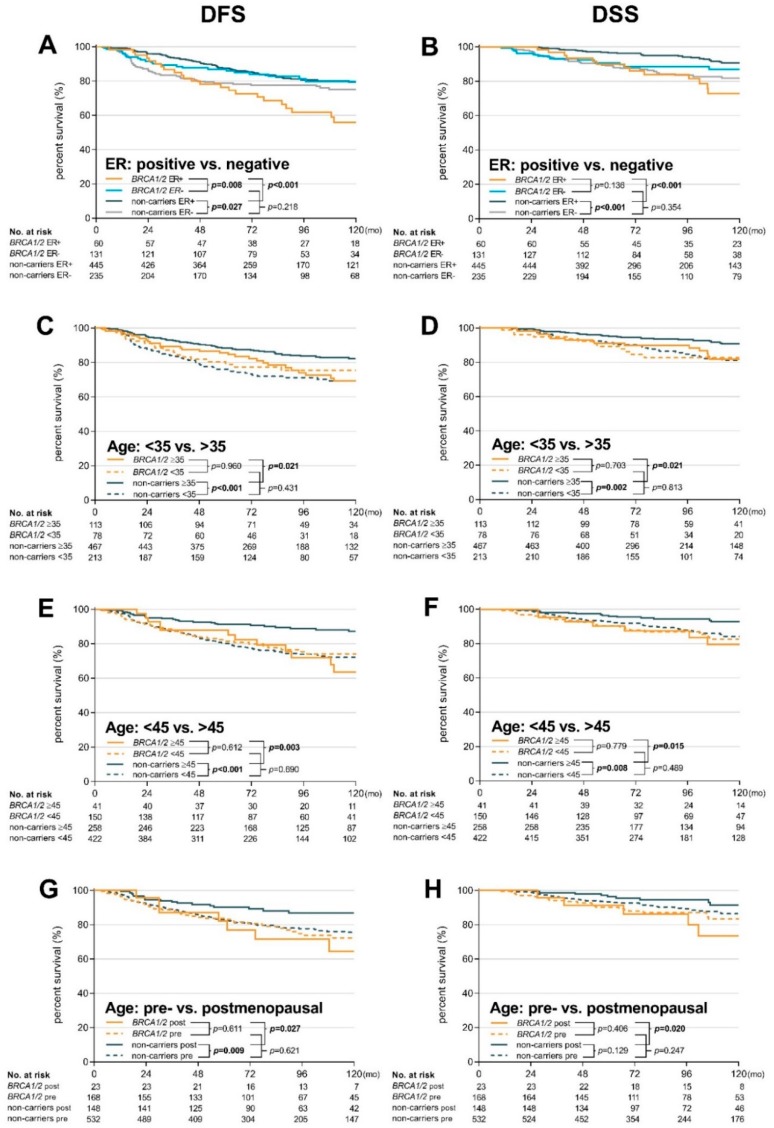
Kaplan–Meier plots of DFS and DSS for: (**A**,**B**) ER status (positive vs. negative); (**C**,**D**) age (<35 vs. ≥35); (**E**,**F**) age (<45 vs. ≥45); and (**G**,**H**) menopausal status (pre- vs. post-menopausal).

To more closely evaluate the opposite effects of age, menopausal status and ER status on survival of *BRCA1/BRCA2* mutation carriers and non-carriers, we plotted Kaplan–Meier curves to visualize the dynamics of survival data from Table 2 and Supplementary Table S3. The survival curves showed that ER-positivity worsened DFS and DSS in *BRCA1/BRCA2* mutation carriers, while the age at diagnosis (positively correlating with the risk) and menopausal status only slightly modified the risk (Figure 2). In contrast, younger age at diagnosis or pre-menopausal status worsened DFS and DSS in non-carriers and ER status only modified the course of survival curves and earlier recurrence in ER-negative patients. Importantly, the negative effect of ER-positivity on survival was comparable between *BRCA1* and *BRCA2* mutation carriers (Figure 1C,D).

Based on these observations, we further analyzed the combined impact of ER-positivity with age at BC onset (or menopausal status). In non-carriers, 10-year DFS and DSS were significantly worsened in younger patients’ subgroups (<35; <45; and premenopausal, respectively) with ER-negative patients relapsing substantially earlier, as expected (Supplementary Figure S3).

In contrast, 10-year DFS was not significantly influenced by age or menopausal status in *BRCA1/BRCA2* mutation carriers, but patients with ER-positive tumors had worsened DFS (with a similar non-significant trend for DSS), compared with patients with ER-negative BC (Supplementary Figure S4). Patients with *BRCA1/BRCA2* mutations diagnosed with ER-positive tumors at ≥35 years showed a significantly increased risk of recurrence (HR 2.53 95% CI 1.15–5.57), compared with ER-negative mutation carriers of the same age. Similarly, increased risk of recurrence was shown for ER-positive patients ≥45 years (HR 4.03 95% CI 1.36–12.00), compared with ER-negative patients of the same age.

A direct comparison of the combined effects of ER status with age at disease onset (or menopausal status) on survival in *BRCA1/BRCA2* mutation carriers and non-carriers is shown in Supplementary Figure S5. *BRCA1/BRCA2* mutation carriers with ER-positive tumors diagnosed at ≥35 years had worse DFS (HR 4.56 95% CI 2.00–10.37) and DSS (HR 8.24 95% CI 2.37–28.72) than ER-positive non-carriers of the same age. Similarly, *BRCA1/BRCA2* mutation carriers with ER-positive tumors diagnosed at ≥45 years or with post-menopausal BC also faced increased risk of recurrence.

The recurrence risk in young (<35 years) ER-negative patients was higher in non-carriers (HR 1.93 95% CI 1.03–3.61; *p* = 0.039) than in *BRCA1/BRCA2* mutation carriers, with the same trend observed for DSS. The non-significant trend was also observed in patients diagnosed at <45 years, but not in premenopausal patients.

**Figure 3:**
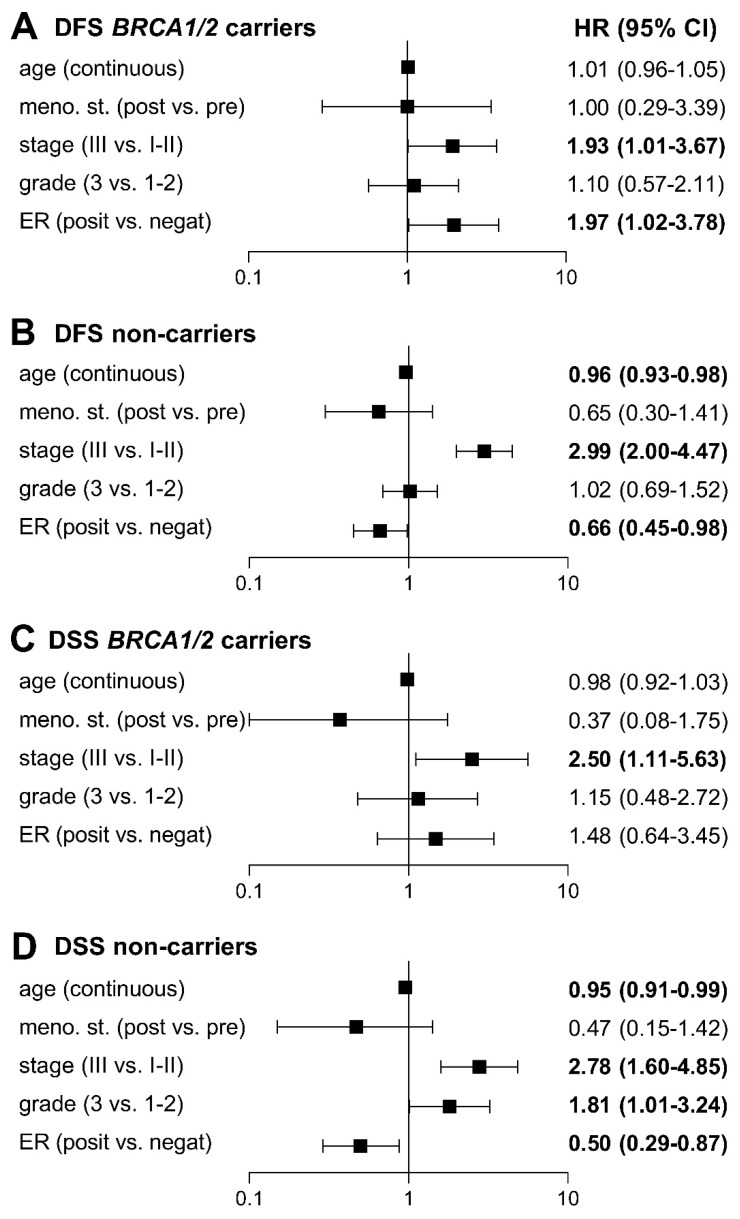
Forest plots of multivariable hazard ratios from model D (Supplementary Table S5) for DFS (**A**,**B**) and DSS (**C**,**D**) in *BRCA1/2* carriers and non-carriers adjusted for age (continuous), menopausal status (post vs. premenopausal), stage (III vs. I–II), grade (3 vs. 1–2), and ER status (positive vs. negative). Bold: statistically significant differences.

To exclude potential bias resulting from differences in baseline clinicopathological characteristics, we analyzed *BRCA1/BRCA2* mutation carriers and non-carriers by multivariable Cox proportional-hazard models considering significantly different covariates from univariate Cox analyses (Supplementary Table S4). The Cox univariate analysis confirmed differences found by the Mantel–Haenszel test (Supplementary Table S5), identifying age at diagnosis, menopausal status, stage, grade, and ER status as statistically significant covariates. We excluded tumor size, nodal and PR status from the multivariable analysis as these covariates directly correlated with tumor stage and ER status. All six models in the multivariable analysis, differing in age (continuous; <35 vs. ≥35 years; <45 vs. ≥45 years) and tumor stage (II–III vs. I; III vs. I–II), confirmed tumor stage as the strongest risk factor (Supplementary Table S5) in both groups, while ER-positivity emerged as a statistically significant negative prognostic factor (with an effect comparable with advanced stage) for recurrence in *BRCA1/BRCA2* mutation carriers. In contrast, ER-positivity reduced the risk in non-carriers (Figure 3). Age at diagnosis was inversely associated with the risk of recurrence in non-carriers only. While ER-positivity non-significantly increased the risk of death in *BRCA1/BRCA2* mutation carriers, it was a strong protective factor in non-carriers. Other variables that negatively affected DSS in non-carriers were only younger age at diagnosis and tumor grade 3.

## 3. Discussion

Our initial analysis revealed slightly worsened DFS and DSS in *BRCA1/BRCA2* mutation carriers compared with non-carriers; however, the difference was less than 10% at 10 years and marginally significant for DSS only (HR = 1.65 95% CI 1.01–2.70). A similar observation was recently reported in a meta-analysis by Baretta and colleagues, showing decreased BC-specific survival (HR = 1.42 95% CI 1.05–1.92) but non-significantly changed DFS for *BRCA1/BRCA2* mutation carriers [18]. A meta-analysis by van den Broek revealed a non-significant trend towards a survival disadvantage for BC outcomes in *BRCA1/BRCA2* mutation carriers [17]. A recent prospective analysis by Copson and colleagues found no significant differences in OS for *BRCA1/BRCA2* mutation carriers [19]. All these data indicate that *BRCA1/BRCA2* mutation carriers have only a slight prognostic disadvantage over non-carriers. However, this conclusion is clinically contra-intuitive because we and others have noted that *BRCA1* mutation carriers (predominating among our mutation-positive patients) are mostly young, TNBC patients who otherwise represent a subpopulation of BC patients with poor prognoses [23,24,25]. This indicates differences between the effects of hormonal receptor status or age at disease onset on *BRCA1/BRCA2* mutation carriers and non-carriers.

Indeed, we observed an inverse correlation between ER status and age in these two groups in initial univariate analyses (Table 2). As expected, the risk of disease recurrence was higher in ER-negative, younger (or premenopausal) non-carriers. Surprisingly, the same was true for ER-positive *BRCA1/BRCA2* mutation carriers who developed disease recurrence 2.3-times more likely (23/60; 38.3%) than ER-positive non-carriers (74/445; 16.6%; *p* < 0.001). ER-positive *BRCA1/BRCA2* mutation carriers also had 3.4-times higher risk of death due to BC compared with ER-positive non-carriers (13/60; 21.7% vs. 28/445; 6.3%; *p* < 0.001). We must emphasize that the prognosis for ER-negative *BRCA1/BRCA2* mutation carriers was comparable with that for ER-positive non-carriers. Inferior survival associated with ER-positive tumors was also reported in a prospective POSH study in *BRCA1* mutation carriers (HR = 1.96 95% CI 1.41–2.71) and *BRCA2* mutation carriers (HR = 2.24 95% CI 1.56–3.22) at 10 years [19]. Interestingly, *BRCA2* ER-positive patients in our study contributed to a worsened prognosis more than *BRCA1* ER-positive patients (Supplementary Figure S2). Jonasson and colleagues showed decreased DSS (HR = 1.61 95% CI 1.11–2.35) in BC patients carrying the Icelandic founder 999del5 *BRCA2* mutation, which was even more pronounced in ER-positive patients (HR = 1.92 95% CI 1.20–3.05) [16]. Schmidt and colleagues reported inferior OS in Dutch ER-positive BC patients with *BRCA2* mutations (HR = 2.04 95% CI 1.22–3.39) but not *BRCA1* mutations [13]. Recent data by Metcalfe and colleagues also found worsened survival in ER-positive BC *BRCA2* mutation carriers (the 20-year survival rate was 62.2% and 83.7% (*p* = 0.03) for ER-positive and ER-negative patients, respectively) [26]. All these data indicate that the prognostic role of ER-positivity differs between *BRCA1/BRCA2* mutation carriers and non-carriers.

Interestingly, Lips and colleagues have shown that ER-positive tumors in both *BRCA1* and *BRCA2* mutation carriers share similar specific genomic profiles of DNA somatic copy number alterations, different from those in ER-positive sporadic tumors and in ER-negative tumors in *BRCA1* mutation carriers [27]. A high number of loss-of-heterozygosity events at the *BRCA1* genomic locus in ER-positive tumors from *BRCA1* mutation carriers found in this study (83%) and in a study by Tung and colleagues (81%) indicates that *BRCA1* impairment directly contributes to the formation of ER-positive tumors [28]. The mechanism explaining how ER signaling can contribute to worsened BC progression in *BRCA1/BRCA2* mutation carriers is unknown; however, preclinical data demonstrated estrogen-dependent progression of mammary tumorigenesis in BRCA1-defficient cells [29,30]. Shah and colleagues have analyzed OncotypeDX in *BRCA1/BRCA2* mutation carriers with ER-positive tumors and found a high proportion of patients with a high recurrence score who may benefit from adjuvant chemotherapy [31].

The age at disease onset negatively correlates with the risk of cancer-related death in unselected BC patients [24,25]. While the negative prognostic effect of younger age was clearly apparent in all age categories for non-carriers also in our study, it did not affect disease recurrence or survival in *BRCA1/BRCA2* mutation carriers (Figure 1). The results of univariate analyses revealed that age at BC onset does not play an important prognostic role in *BRCA1/BRCA2* mutation carriers and indicated that age should not be considered as a factor influencing patients’ treatment approaches. This observation will require further evaluation in larger cohorts because carriers of *BRCA1/BRCA2* mutation develop BC significantly earlier than non-carriers and many published studies focused primarily on early-onset BC patients or had enriched this subgroup as a result of criteria for genetic testing [10,13,19].

The multivariable analysis confirmed an inverse prognostic effect of ER-positivity and the age at disease onset on *BRCA1/BRCA2* mutation carriers against non-carriers. The HR in ER-positive patients for disease recurrence was 1.97 (95% CI 1.02–3.78) in *BRCA1/BRCA2* carriers and 0.66 (95% CI 0.45–0.98) in non-carriers with a similar trend for survival (Figure 3). The multivariable analysis also showed a significant age-dependent decrease in risk of recurrence and cancer-related death in non-carriers but not in *BRCA1/BRCA2* carriers.

Hormone receptor-positive BC (regardless of the *BRCA1/BRCA2* mutation status) is currently considered a cancer with a favorable prognosis, allowing the omission of adjuvant chemotherapy, shorter course of adjuvant hormonal treatment (no longer than five years), and other “de-escalation” approaches. In contrast, our data suggest that ER-positive *BRCA1/BRCA2* mutation carriers exert an extremely dismal prognosis. We suppose that this patient group should be considered a high-risk group for BC recurrence and BC-related death.

Our study has several strengths. All genotyping was done in a single center using systematic counseling and testing criteria. We analyzed a homogenous set of BC patients excluding patients with breast and ovarian/pancreatic cancer duplicity and patients carrying mutations in non-*BRCA1/BRCA2* BC-susceptibility genes. Furthermore, data were highly consistent as only 18.4% of *BRCA1/BRCA2* carriers and 24.4% of non-carriers (mainly those enrolled before 2005) were excluded due to incomplete data (a proportion comparable with a prospective POSH trial) [19]. We used DSS instead of OS (to exclude death events from non-BC causes) for more accurate survival analyses. Study limitations included a limited sample size; however, only four out of 66 studies evaluated in a meta-analysis by van den Broek and colleagues surpassed the number of 191 mutation carriers analyzed in our study [17]. A subsequent meta-analysis of *BRCA1/BRCA2* mutation carriers’ prognoses by Baretta and colleagues identified 60 studies and revealed the median number of *BRCA1/BRCA2* mutation carriers as 39.5 (range 5–326). The size limitation affected especially *BRCA2* mutation carriers (representing a minority population compared with *BRCA1* mutation carriers in the Czech Republic [4]) and *BRCA1* mutation carriers with ER-positive BC (representing 22.5% of all *BRCA1* mutation carriers in our study). Further limitations resulted from the study design and a retrospective character of data. We performed a two-round, independent review of all clinicopathological data (irrespectively to the *BRCA1/BRCA2* mutation status) obtained from the medical records. However, we cannot exclude a potential selection bias as 23.2% of all enrolled patients were excluded due to incomplete data from the univariate and multivariable analyses. On the other hand, no patient was excluded due to the loss of follow-up from the survival analyses. Retrospective study design is also sensitive to changes made in diagnostics and treatment procedures during the 19-year study period (1997–2015). Histopathological assessments for ER, PR, and HER-2 positivity have not been identical for all patients throughout the study period. Potential bias represent changes in BC treatment guidelines (especially for chemotherapy administration) during the study period, including the introduction of taxanes and trastuzumab, routinely available in the last 15 years. We are also aware that a further extension of the median follow-up (currently 9.8 years) will be necessary to better evaluate the effect of ER-positivity on DSS. Future studies screening *BRCA1/BRCA2* mutations in an unselected BC population prospectively should be conducted to further examine differences in clinicopathological characteristics in *BRCA1/BRCA2*-positive and *BRCA1/BRCA2*-negative patients in the general BC population.

## 4. Materials and Methods

### 4.1. Patient Characteristics

We enrolled 1133 unrelated, female BC patients (including 234 *BRCA1/BRCA2* mutation carriers and 899 non-carriers) who were tested for the presence of mutations in *BRCA1/BRCA2* and other cancer-susceptibility genes at the Laboratory of Oncogenetics, First Faculty of Medicine, Charles University, in 1997–2015. All patients met national criteria for genetic/familial high-risk assessment in BC/OC (Supplementary Table S6) to be eligible for genetic counseling and testing. Patients with duplicity of breast and ovarian/pancreatic cancer were not enrolled because of the extensive impact on cancer prognosis. We also excluded a heterogeneous group of patients carrying mutations in non-*BRCA1/BRCA2* BC/OC-susceptibility genes. Clinicopathological data (Supplementary Table S2) were retrieved from clinical documentation and independently reviewed by a two-round evaluation (last assessed November, 2018). All patients were Caucasians of Czech origin. The study was approved by the Ethics Committee of the General University Hospital, Prague (ethic code:1858/14 S-IV).

### 4.2. Molecular Analysis

An analysis of mutations in BC-susceptibility genes was initially performed using a protein truncation test or direct sequencing, the presence of large genomic *BRCA1/BRCA2* rearrangements was analyzed by multiplex ligation-dependent probe amplification (MRC Holland, Amsterdam, the Netherlands), as described previously [32,33,34]. As of 2015, all samples were analyzed using the custom-designed CZECANCA panel (NimbleGen/Roche, Pleasanton, CA, USA) targeting 219 cancer-susceptible genes on MiSeq (Illumina, San Diego, CA, USA) [35]. The bioinformatics analysis included the identification of pathogenic mutations (single nucleotide variants described as pathogenic in ClinVar, non-sense, frame-shift, splicing-site alterations, and copy number variants) using a pipeline described recently [35,36].

### 4.3. Statistical Methods

Categorical variables (including age, menopausal status, tumor stage, tumor size, nodal status, morphology, tumor grade, ER, PR, HER-2, BC subtypes, surgery, radiotherapy, chemotherapy, endocrine therapy, event during follow-up, median follow-up, and death due to BC) were compared between *BRCA1/BRCA2* mutation carriers and non-carriers using χ^2^ or Fisher exact tests, where appropriate. Continuous variables (age at diagnosis and follow-up period) were tested by the Mann–Whitney test.

The Kaplan–Meier product-limit method was used for survival analyses and differences were tested using the log-rank and Mantel–Haenszel tests. BC patients with carcinoma in situ (*N* = 46) or primarily metastatic BC (*N* = 25) were excluded from survival analyses. DFS was defined as the interval between BC diagnosis and the first loco-regional or distant recurrence or the last follow-up. The development of a second tumor was not considered a DFS event. DSS was defined as the interval between BC diagnosis and death from BC or the last follow-up.

Univariate analyses of categorical variables (age, menopausal status, stage, grade, ER, PR, HER-2 status, and TNBC) and multivariate analyses (age, menopausal status, stage, grade, and ER status) were performed using Cox proportional hazard regression.

All analyses were performed using the GraphPad Prism v8.0.1 (GraphPad Software, San Diego, CA, USA) and Statistica v12 (StatSoft, Palo Alto, CA, USA) programs. Two-sided *p* values < 0.05 and 95% confidence intervals (CI) excluding 1 were considered statistically significant.

## 5. Conclusions

The present study indicates that *BRCA1/BRCA2* mutation carriers with ER-positive tumors have a poor prognosis with increased BC recurrence and BC-related death rate; therefore, their specific treatment (surgical and pharmacological prevention) should be considered. The BC prognosis for these patients is worse than that for young ER-negative BC non-carriers. In contrast, the prognosis for *BRCA1/BRCA2* mutation carriers with ER-negative tumors, even with early BC onset, is comparable with ER-positive, older BC non-carriers, who are generally considered lower-risk patients.

## Figures and Tables

**Table 1:** Clinicopathological characteristics of *BRCA1/BRCA2* mutation carriers (together and separately) and non-carriers of mutations in cancer-susceptibility genes for whom complete clinicopathological data were available for subsequent univariate and multivariable analyses.

	*BRCA1/2* Carriers (*N* = 191)			*BRCA1* Carriers (*N* = 151)			*BRCA2* Carriers (*N* = 40)			Non-Carriers (*N* = 680)		
	*N*	%*	*p*	*N*	%*	*P*	*N*	%*	*p*	*N*	%*	*p*
Median age at diagnosis												
year (25–75% percentile)	37.1 (32.3–43.8)		**<0.001**	36.9 (31.9–42.6)		**<0.001**	38.7 (32.9–50.8)		0.645	40.2 (33.5–49.6)		Ref.
Age diagnosis categories (known)												
<35 years	78	(40.8)	**<0.001**	64	(42.4))	**<0.001**	14	(35.0)	0.601	213	(31.3)	Ref.
35–44 years	72	(37.7)		58	(38.4)		14	(35.0)		209	(30.7)	
≥45 years	41	(21.5)		29	(19.2)		12	(30.0)		258	(38.0)	
Menopausal status												
Pre	168	(88.0)	**0.003**	136	(90.1)	**<0.001**	32	(80.0)	0.792	532	(78.2)	Ref.
post	23	(12.0)		15	(9.9)		8	(20.0)		148	(21.8)	
Primary tumor (T)												
T1 (<2 cm)	71	(37.2)	**0.001**	52	(34.4)	**<0.001**	19	(47.5)	0.093	355	(52.2)	Ref.
T2 (2–5 cm)	84	(44.0)		73	(48.3)		11	(27.5)		244	(35.9)	
T3 (>5 cm)	24	(12.6)		17	(11.3)		7	(17.5)		62	(9.1)	
T4	12	(6.3)		9	(6.0)		3	(7.5)		19	(2.8)	
Regional lymphatic node (N)												
N0	100	(52.4)	0.296	85	(56.3)	0.206	15	(37.5)	0.077	387	(56.9)	Ref.
N1	78	(40.8)		55	(36.4)		23	(57.5)		256	(37.6)	
N2	8	(4.2)		6	(4.0)		2	(5.0)		30	(4.4)	
N3	5	(2.6)		5	(3.3)		0	(0.0)		7	(1.0)	
Tumor stage												
I (T1N0–1mi)	52	(27.2)	**<0.001**	41	(27.2)	**0.002**	11	(27.5)	**0.022**	282	(41.5)	Ref.
II (T2–3N0, T1–2N1)	97	(50.8)		79	(52.3)		18	(45.0)		310	(45.6)	
III (T3N1, TXN2–3, T4NX)	42	(22.0)		31	(20.5)		11	(27.5)		88	(12.9)	
Breast tumor morphology												
ductal	168	(88.0)	**0.023**	131	(86.8)	**0.005**	37	(92.5)	0.325	574	(84.4)	Ref.
lobular	6	(3.1)		3	(2.0)		3	(7.5)		52	(7.6)	
medullar	15	(7.9)		15	(9.9)		0	(0.0)		33	(4.9)	
other	2	(1.0)		2	(1.3)		0	(0.0)		21	(3.1)	
Grade												
low (1)	7	(3.7)	**<0.001**	4	(2.6)	**<0.001**	3	(7.5)	0.554	91	(13.4)	Ref.
intermediate (2)	59	(30.9)		40	(26.5)		19	(47.5)		311	(45.7)	
high (3)	125	(65.4)		107	(70.9)		18	(45.0)		278	(40.9)	
ER status												
positive	60	(31.4)	**<0.001**	34	(22.5)	**<0.001**	26	(65.0)	>0.99	445	(65.4)	Ref.
PR status												
positive	61	(31.9)	**<0.001**	34	(22.5)	**<0.001**	27	(67.5)	0.616	428	(62.9)	Ref.
HER-2 status												
positive	13	(6.8)	**<0.001**	9	(6.0)	**<0.001**	4	(10.0)	0.052	164	(24.1)	Ref.
TNBC												
yes	114	(59.7)	**<0.001**	105	(69.5)	**<0.001**	9	(22.5)	0.690	138	(20.3)	Ref.
Surgery—primary tumor												
mastectomy	91	(47.6)	0.774	70	(46.4)	0.980	21	(52.5)	0.458	316	(46.5)	Ref.
breast-conserving surgery	100	(52.4)		81	(53.6)		19	(47.5)		364	(53.5)	
Surgery—lymphatic nodes												
axillary dissection	149	(78.0)	0.051	117	(77.5)	0.102	32	(80.0)	0.215	482	(70.9)	Ref.
sentinel node biopsy	42	(22.0)		34	(22.5)		8	(20.0)		198	(29.1)	
Radiotherapy												
yes	132	(69.1)	0.759	108	(71.5)	0.391	24	(60.0)	0.297	462	(67.9)	Ref.
Chemotherapy type												
Antra + Tax	122	(63.9)	**<0.001**	97	(64.2)	**<0.001**	25	(62.5)	0.096	308	(45.3)	Ref.
Antra	50	(26.2)		39	(25.8)		11	(27.5)		195	(28.7)	
Other	7	(3.7)		6	(4.0)		1	(2.5)		32	(4.7)	
No chemotherapy	12	(6.3)		9	(6.0)		3	(7.5)		145	(21.3)	
Endocrine therapy **												
TAM monotherapy	21	(35.0)	0.951	14	(41.2)	0.947	7	(26.9)	0.745	165	(37.1)	Ref.
AI monotherapy	8	(13.4)		4	(11.8)		4	(15.4)		65	(14.6)	
LHRH analogues + TAM	29	(48.3)		15	(44.1)		14	(53.8)		204	(45.8)	
LHRH analogues + AI	2	(3.3)		1	(2.9)		1	(3.9)		11	(2.5)	
Event during follow-up ***												
loco-regional recurrence	5	(2.6)	**0.036**	5	(3.3)	0.122	0	(0.0)	-	45	(6.6)	Ref.
distant metastasis	41	(21.5)	**0.001**	25	(16.6)	0.150	16	(40.0)	**<0.001**	83	(12.2)	Ref.
second breast cancer	24	(12.6)	**0.009**	34	(22.5)	**<0.001**	9	(22.5)	**<0.001**	46	(6.8)	Ref.
second tumors	7	(3.7)	0.322	10	(6.6)	0.570	2	(5.0)	0.905	37	(5.4)	Ref.
Median of follow-up												
median (25–75% percentile)	8.6 (6.0–12.1)		0.235	8.2 (5.7–11.8)		0.733	9.4 (6.9–13.4)		0.031	8.2 (5.6–11.8)		Ref.
Breast cancer related death												
yes	28	(14.7)	0.507	16	(10.6)	0.655	12	(30.0)	**<0.001**	64	(9.4)	Ref.

* % = percentage of known; ** *N* = number of patients with ER-positive BC; *** patient could be counted in more than one event. pre—premenopausal; post—postmenopausal; TNBC—triple-negative BC; Antra—anthracyclines; Tax—taxanes; TAM—tamoxifen; AI—aromatase inhibitor; LHRH—luteinizing hormone-releasing hormone; Ref—reference. Bold: statistically significant differences.

**Table 2:** Analysis of 10-year DFS and DSS using the Mantel–Haenszel test comparing variables between *BRCA1/2* mutation carriers and non-carriers.

		Disease Free Survival (DFS) Analysis										Disease Specific Survival (DSS) Analysis									
		*BRCA1/2* Carriers						Non-Carriers				*BRCA1/2* Carriers						Non-Carriers			
		Pts No.	Ev No.	Ev %	HR	95% CI	*p*	Pts No.	Ev No.	Ev %		Pts No.	Ev No.	Ev %	HR	95% CI	*p*	Pts No.	Ev No.	Ev %	
**All pts**		191	46	24.1	1.29	0.90–1.84	0.166	680	128	18.8	Ref.	191	28	14.7	**1.65**	**1.01–2.70**	**0.047**	680	64	9.4	Ref.
**Age at diagnosis**	<35	78	18	23.1	0.82	0.50–1.35	0.431	213	60	28.2	Ref.	78	12	15.4	1.09	0.55–2.13	0.813	213	32	15.0	Ref.
	≥35	113	28	24.8	**1.81**	**1.10–2.99**	**0.021**	467	68	14.6	Ref.	113	16	14.2	**2.29**	**1.13–4.65**	**0.021**	467	32	6.9	Ref.
	<45	150	34	22.7	0.93	0.63–1.36	0.690	422	100	23.7	Ref.	150	21	14.0	1.21	0.71–2.05	0.489	422	49	11.6	Ref.
	≥45	41	12	29.3	**3.98**	**1.62–9.81**	**0.003**	258	28	10.9	Ref.	41	7	17.1	**4.48**	**1.34–15.0**	**0.015**	258	15	5.8	Ref.
**Menopausal status**	pre	168	39	23.2	1.10	0.76–1.60	0.621	532	119	22.4	Ref.	168	23	13.7	1.36	0.81–2.29	0.247	532	55	10.3	Ref.
	post	23	7	30.4	**3.72**	**1.16–11.9**	**0.027**	148	18	12.2	Ref.	23	5	21.7	**5.95**	**1.32–26.8**	**0.020**	148	9	6.1	Ref.
**Tumor size**	T1	71	11	15.5	1.23	0.61–2.47	0.569	355	45	12.7	Ref.	71	4	5.6	1.13	0.37–3.74	0.835	355	18	5.1	Ref.
	T2	84	20	23.8	1.07	0.63–1.81	0.802	244	52	21.3	Ref.	84	12	14.3	1.17	0.58–2.36	0.653	244	29	11.9	Ref.
	T3	24	9	37.5	0.90	0.42–1.92	0.787	62	23	37.1	Ref.	24	7	29.2	1.30	0.50–3.41	0.594	62	13	21.0	Ref.
	T4	12	6	50.0	1.42	0.47–4.29	0.530	19	8	42.1	Ref.	12	5	41.7	2.22	0.57–8.62	0.249	19	4	21.1	Ref.
**Nodal status**	N0	100	17	17.0	1.26	0.70–2.26	0.435	387	51	13.2	Ref.	100	11	11.0	**2.55**	**1.06–6.12**	**0.037**	387	19	4.9	Ref.
	N1	78	25	32.1	1.37	0.83–2.25	0.217	256	64	25.0	Ref.	78	15	19.2	1.42	0.74–2.72	0.285	256	38	14.8	Ref.
	N2	8	2	25.0	0.72	0.18–2.90	0.647	30	9	30.0	Ref.	8	1	12.5	0.86	0.11–7.02	0.890	30	4	13.3	Ref.
	N3	5	2	40.0	0.65	0.13–3.30	0.605	7	4	57.1	Ref.	5	1	20.0	0.40	0.05–3.07	0.382	7	3	42.9	Ref.
**Tumor stage**	I	52	7	13.5	1.42	0.57–3.54	0.457	282	28	9.9	Ref.	52	2	3.8	1.35	0.25–7.31	0.730	282	8	2.8	Ref.
	II	97	23	23.7	1.09	0.67–1.77	0.733	310	65	21.0	Ref.	97	15	15.5	1.26	0.67–2.37	0.472	310	37	11.9	Ref.
	III	42	16	38.1	0.89	0.50–1.60	0.699	88	35	39.8	Ref.	42	11	26.2	1.22	0.57–2.63	0.608	88	19	21.6	Ref.
**Tumor grade**	1	7	2	28.6	9.83	0.77–125.4	0.079	91	6	6.6	Ref.	7	1	14.3	-	-	-	91	1	1.1	Ref.
	2	59	16	27.1	1.44	0.78–2.66	0.240	311	59	19.0	Ref.	59	9	15.3	**2.71**	**1.03–7.13**	**0.044**	311	21	6.8	Ref.
	3	125	28	22.4	0.96	0.61–1.49	0.841	278	63	22.7	Ref.	125	18	14.4	0.94	0.54–1.62	0.824	278	42	15.1	Ref.
**ER status**	pos	60	23	38.3	**3.14**	**1.69–5.81**	**<0.001**	445	74	16.6	Ref.	60	13	21.7	**5.70**	**2.27–14.4**	**<0.001**	445	28	6.3	Ref.
	neg	131	23	17.6	0.75	0.47–1.19	0.218	235	54	23.0	Ref.	131	15	11.5	0.76	0.43–1.35	0.354	235	36	15.3	Ref.
**PR status**	pos	62	22	35.5	**2.52**	**1.38–4.60**	**0.003**	428	76	17.8	Ref.	62	10	16.1	**2.85**	**1.44–7.14**	**0.026**	428	30	7.0	Ref.
	neg	129	24	18.6	0.87	0.54–1.40	0.566	252	52	20.6	Ref.	129	18	14.0	1.02	0.57–1.80	0.954	252	34	13.5	Ref.
**HER-2 status**	pos	13	4	30.8	1.68	0.49–5.78	0.413	164	34	20.7	Ref.	13	2	15.4	1.44	0.27–7.60	0.668	164	19	11.6	Ref.
	neg	129	24	18.6	0.87	0.54–1.40	0.566	252	52	20.6	Ref.	129	18	14.0	1.02	0.57–1.80	0.954	252	34	13.5	Ref.
**TNBC**	yes	114	20	17.5	0.77	0.44–1.35	0.367	138	30	21.7	Ref.	114	14	12.3	0.78	0.40–1.53	0.475	138	21	15.2	Ref.
	no	77	26	33.8	**2.27**	**1.32–3.88**	**0.003**	542	98	18.1	Ref.	77	14	18.2	**2.92**	**1.34–6.38**	**0.007**	542	43	7.9	Ref.

Pts No.—number of patients; Ev No.—number of events; Ev %—percentage of events; HR—hazard ratio; 95% CI—95% confidential interval; pre—premenopausal; post—postmenopausal; pos—positive; neg—negative; Ref.—reference. Bold: statistically significant differences.

## Supplementary Materials

The following are available online at https://www.mdpi.com/2072-6694/11/6/738/s1, Figure S1: Differences in the age at diagnosis in mutation carriers and non-carriers in particular breast cancer subtypes, Figure S2: Kaplan–Meier plots showing DFS and DSS in *BRCA1* mutation carriers, *BRCA2* mutation carriers, and non-carriers, Figure S3: Kaplan–Meier plots of DFS and DSS in *BRCA1/2* mutations non-carriers considering a combined impact of ER-status and age at onset or menopausal status on survival, Figure S4: Kaplan–Meier plots of DFS and DSS in *BRCA1/2* mutation carriers considering a combined impact of ER-status and age at onset or menopausal status on survival, Figure S5. Kaplan–Meier plots of DFS and DSS in *BRCA1/2* mutation carriers and non-carriers considering a combined effect of ER-positivity or ER-negativity and age at disease onset or menopausal status on survival, Table S1: List of pathogenic mutations identified in the *BRCA1* and *BRCA2* genes in analyzed breast cancer patients, Table S2: Clinicopathological characteristics of all *BRCA1/BRCA2* mutation carriers (together and separately) and non-carriers of mutations in cancer-susceptibility genes, Table S3: Analysis of 10-year DFS and DSS using the Mantel–Haenszel test comparing variables within subgroups of *BRCA1/2* mutation carriers and non-carriers, respectively, Table S4: Analysis of 10-year DFS and DSS using a univariate Cox regression model comparing variables within subgroups of *BRCA1/2* mutation carriers and non-carriers, Table S5: Analysis of 10-year DFS and DSS using multivariable Cox proportional-hazard models adjusted for age, menopausal status, stage, tumor grade and ER status, Table S6: The indication criteria for genetic testing of hereditary BC/OC predisposition in the Czech Republic.
