# Supplementary material for: Estrogen Receptor Status Oppositely Modifies Breast Cancer Prognosis in BRCA1/BRCA2 Mutation Carriers Versus Non-Carriers
Source: Cancers (Basel). 2019 May 28;11(6):738. doi: 10.3390/cancers11060738 (PMC6627684; doi:10.3390/cancers11060738)
Supplement: Supplementary file 1 [file cancers-11-00738-s001.pdf]

cancers

MDPI

Supplementary Materials: Estrogen Receptor Status Oppositely Modifies Breast Cancer Prognosis in *BRCA1*/*BRCA2* Mutation Carriers Versus Non-Carriers

Michal Vocka, Martina Zimovjanova, Zuzana Bielcikova, Petra Tesarova, Lubos Petruzelka, Martin Mateju, Ludmila Krizova, Jaroslav Kotlas, Jana Soukupova, Marketa Janatova, Petra Zemankova, Petra Kleiblova, Jan Novotny, Bohuslav Konopasek, Martina Chodacka, Milan Brychta, Marek Sochor, Denisa Smejkalova-Musilova, Vlastimila Cmejlova, Renata Kozevnikovova, Lenka Miskarova, Sona Argalacsova, Lenka Stolarova, Klara Lhotova, Marianna Borecka and Zdenek Kleibl

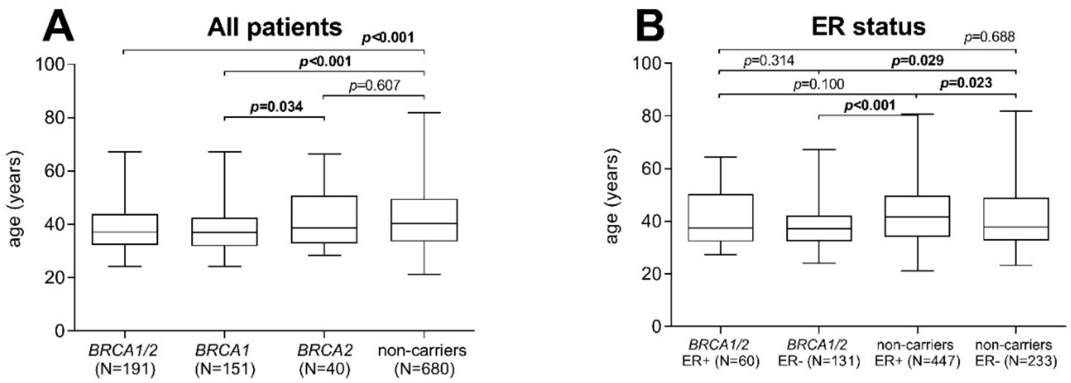

Figure S1. Differences in the age at diagnosis in mutation carriers and non-carriers in particular breast cancer subtypes; (**A**) distribution according to the mutation status; (**B**) distribution according to the mutation status and ER status.

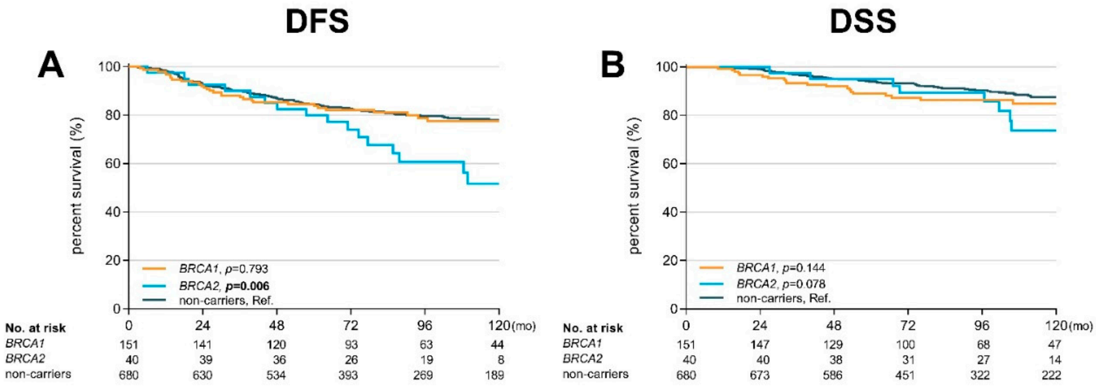

Figure S2. Kaplan–Meier plots showing DFS (**A**) and DSS (**B**) in *BRCA1* mutation carriers, *BRCA2* mutation carriers, and non-carriers.

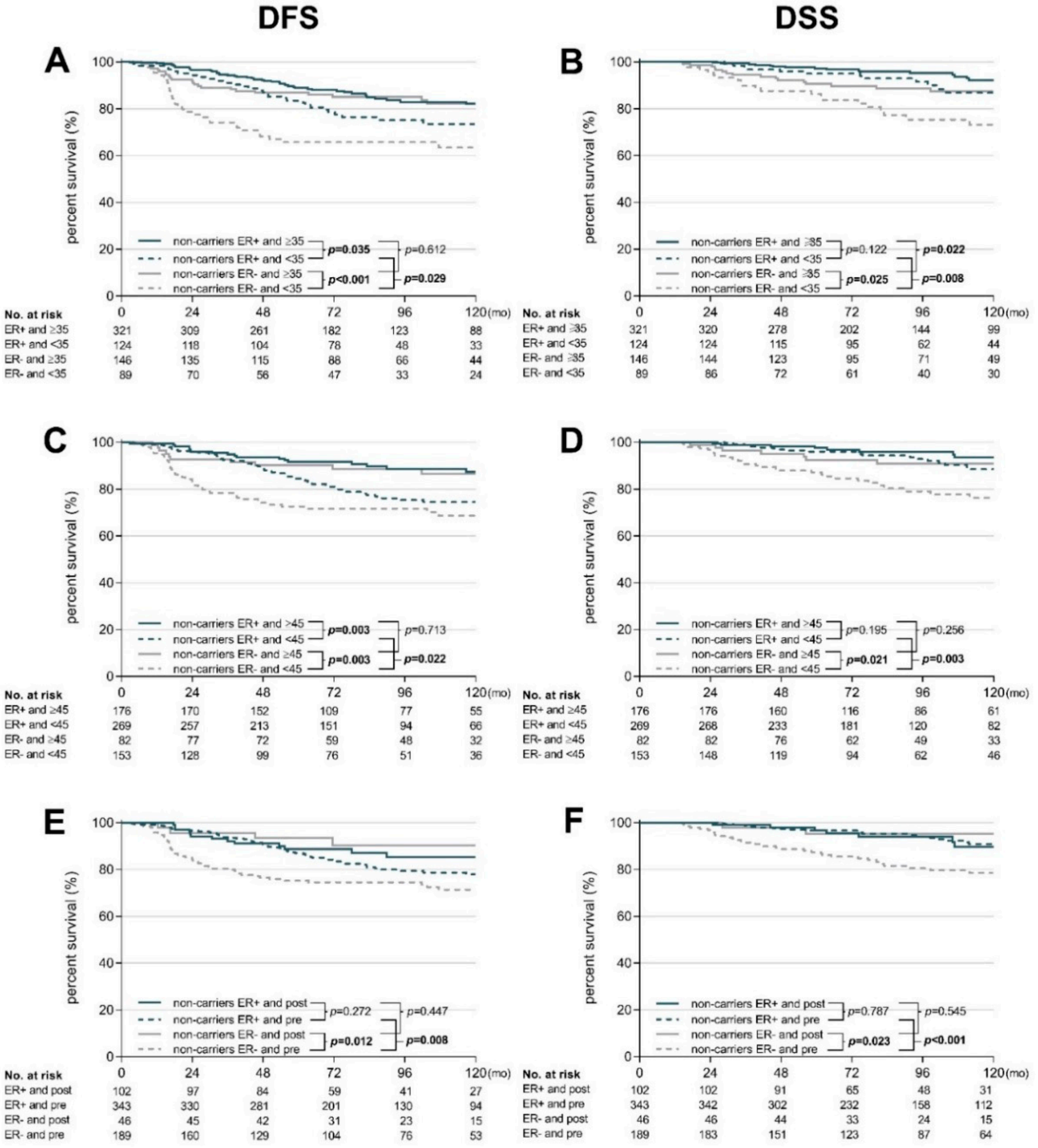

Figure S3. Kaplan–Meier plots of DFS and DSS in *BRCA1/2* mutations non-carriers considering a combined impact of ER-status and age of onset (**A**, **B** <35 vs. ≥35; **C**, **D** <45 vs. ≥45) or menopausal status (**E**, **F**) on survival. (**A**) ER-negative patients diagnosed at <35 years were at higher risk of recurrence (HR 2.61 [95%CI 1.49–4.56]) than ER-negative women diagnosed at ≥35 years. These high-risk ER-negative young patients (diagnosed at <35 years) were also at higher risk (HR 1.79 [95%CI 1.06–3.02]) than ER-positive young women diagnosed at the same age. (**B**) ER-negative patients diagnosed at <35 years were at higher risk of BC-related death than ER-negative women diagnosed at ≥35 years (HR 2.17 [95%CI 1.10–4.25]) and also than ER-positive young women diagnosed at the same age (HR 2.61 [95%CI 1.29–5.30]). (**C**) ER-negative patients diagnosed at <45 years were at higher risk of recurrence than ER-negative women diagnosed at ≥45 years (HR 2.29 [95%CI 1.32–3.98]) and also than ER-positive young women diagnosed at the same age (HR 1.63 [95%CI 1.07–2.47]). (**D**) ER-negative patients diagnosed at <45 years were at higher risk of BC-related death than ER-negative women diagnosed at ≥45 years (HR 2.22 [95%CI 1.13–4.35]) and also than ER-positive young women diagnosed at the same age (HR 3.00 [95%CI 1.67–5.42]). (**E**) Besides, ER-negative premenopausal patients were at higher risk than ER-negative postmenopausal patients (HR 2.31 [95%CI 1.20–4.42]) and ER-positive premenopausal patients (HR 1.71 [95%CI 1.15–2.53]). (**F**) ER-negative premenopausal patients were at higher risk than ER-negative postmenopausal patients (HR 2.53 [95%CI 1.14–5.66]) and ER-positive premenopausal patients (HR 3.36 [95%CI 1.93–5.87]).

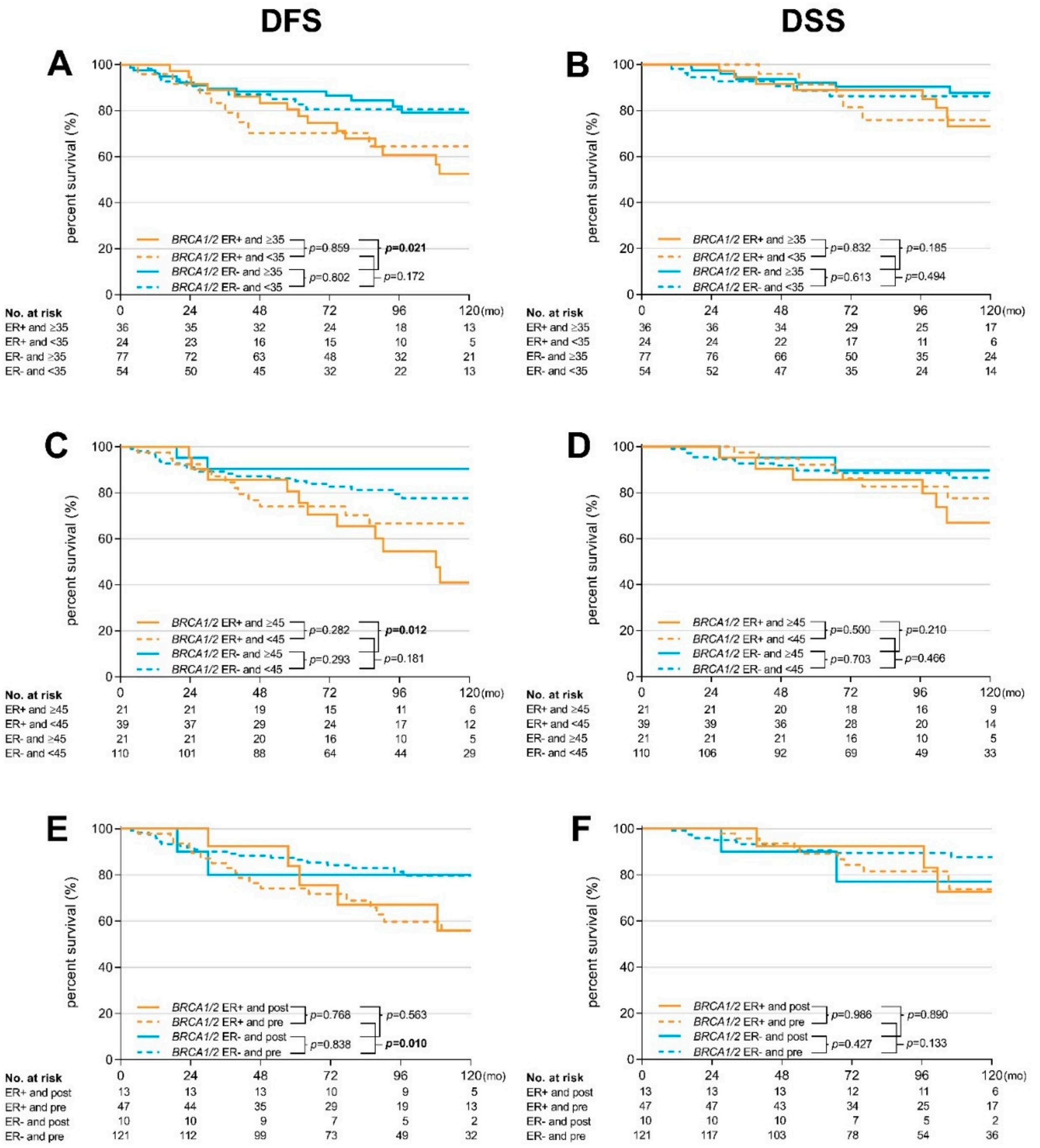

Figure S4. Kaplan–Meier plots of DFS and DSS in *BRCA1/2* mutation carriers considering a combined impact of ER-status and age of onset (**A**, **B** <35 vs. ≥35; **C**, **D** <45 vs. ≥45) or menopausal status (**E**, **F**) on survival. (**A**) Patients diagnosed at ≥35 years with ER-positive tumors showed a significantly increased risk of BC recurrence (HR 2.53 [95%CI 1.15–5.57]), compared with ER-negative mutation carriers of the same age. (**B**) Risk of BC-related death in ER-positive patients diagnosed at ≥35 years did not significantly differ from ER-negative women diagnosed at ≥35 years (HR 2.01 [95%CI 0.72–5.66]). (**C**) Increased risk of BC recurrence was shown for ER-positive patients ≥45 years (HR 4.03 [95%CI 1.36–12.00]), compared with ER-negative patients of the same age. (**D**) Risk of BC-related death in ER-positive patients diagnosed at ≥45 years did not significantly differ from ER-negative women diagnosed at ≥45 years (HR 2.44 [95%CI 0.60–9.85]). (**E**) ER-positive postmenopausal patients were at similar risk of BC recurrence as ER-negative postmenopausal patients (HR 1.57 [95%CI 0.34–7.15]). (**F**) Risk of BC-related death in ER-positive postmenopausal patients did not significantly differ from ER-negative postmenopausal patients (HR 0.88 [95%CI 0.14–5.46]).

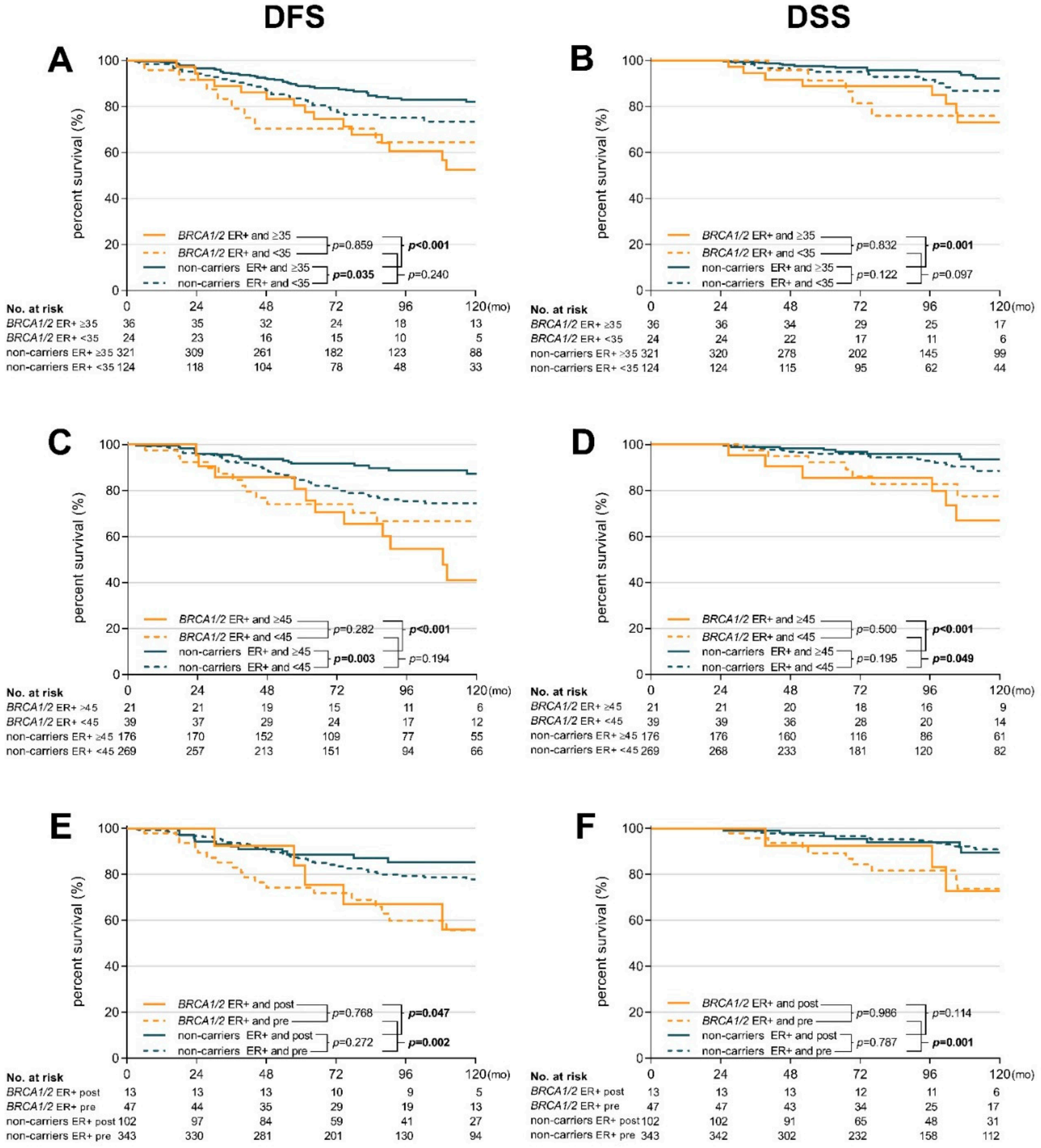

Figure S5. *Cont.*

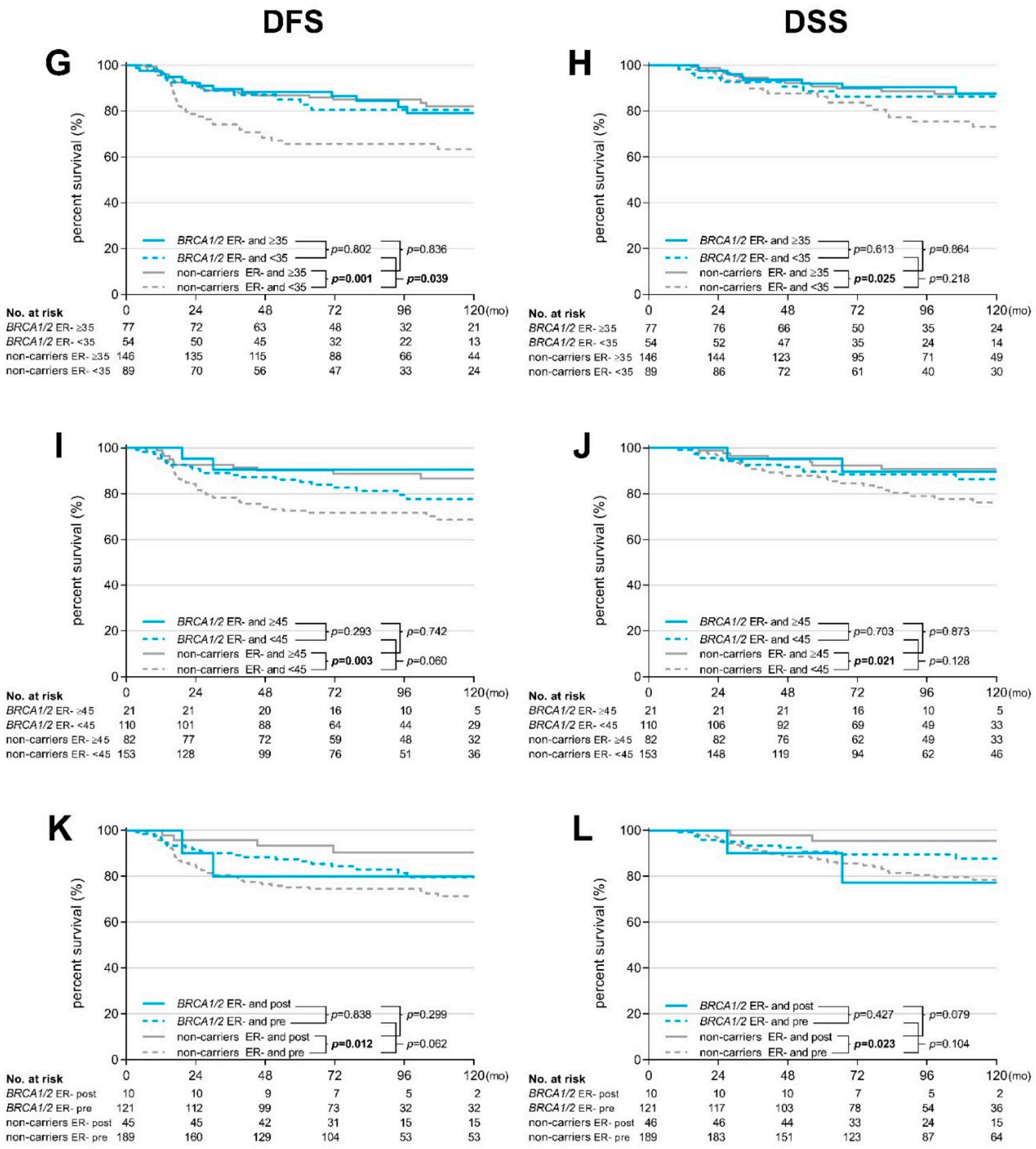

No. at risk (G)	0	24	48	72	96	120 (mo)
BRCA1/2 ER- ≥35	77	72	63	48	32	21
BRCA1/2 ER- <35	54	50	45	32	22	13
non-carriers ER- ≥35	146	135	115	88	66	44
non-carriers ER- <35	89	70	56	47	33	24

No. at risk (H)	0	24	48	72	96	120 (mo)
BRCA1/2 ER- ≥35	77	76	66	50	35	24
BRCA1/2 ER- <35	54	52	47	35	24	14
non-carriers ER- ≥35	146	144	123	95	71	49
non-carriers ER- <35	89	86	72	61	40	30

No. at risk (I)	0	24	48	72	96	120 (mo)
BRCA1/2 ER- ≥45	21	21	20	16	10	5
BRCA1/2 ER- <45	110	101	88	64	44	29
non-carriers ER- ≥45	82	77	72	59	48	32
non-carriers ER- <45	153	128	99	76	51	36

No. at risk (J)	0	24	48	72	96	120 (mo)
BRCA1/2 ER- ≥45	21	21	21	16	10	5
BRCA1/2 ER- <45	110	106	92	69	49	33
non-carriers ER- ≥45	82	82	76	62	49	33
non-carriers ER- <45	153	148	119	94	62	46

No. at risk (K)	0	24	48	72	96	120 (mo)
BRCA1/2 ER- post	10	10	9	7	5	2
BRCA1/2 ER- pre	121	112	99	73	32	32
non-carriers ER- post	45	45	42	31	15	15
non-carriers ER- pre	189	160	129	104	53	53

No. at risk (L)	0	24	48	72	96	120 (mo)
BRCA1/2 ER- post	10	10	10	7	5	2
BRCA1/2 ER- pre	121	117	103	78	54	36
non-carriers ER- post	46	46	44	33	24	15
non-carriers ER- pre	189	183	151	123	87	64

Figure S5. Kaplan–Meier plots of DFS and DSS in *BRCA1/2* mutation carriers and non-carriers considering a combined effect of ER-positivity (**A**–**F**) or ER-negativity (**G**–**L**) and age of disease onset or menopausal status on survival.

Table S1. List of pathogenic mutations identified in the *BRCA1* and *BRCA2* genes in analyzed breast cancer patients.

***BRCA1*/2 Mutation Carries**					
**Gene**	**Mutation**	**No.**	**Gene**	**Mutation**	**No.**
BRCA1	c.5266dupC	73	***BRCA2***	c.5682C>G	4
	c.181T>G	15		c.3847_3848delGT	3
	c.3700_3704delGTAAA	14		c.5946_5946delT	3
	c.1687C>T	9		c.7913_7917delTTCCT	3
	c.213-12A>G	5		c.2808_2811delACAA	2
	c.66_67delAG	5		c.4284_4285insT	2
	c.2411_2412delAG	4		c.7595_7596insTT	2
	c.3756_3759delGTCT	4		c.8042_8043delCA	2
	c.1600C>T	2		c.8755-1G>A	2
	c.3016_3019delCATT	2		c.9097_9098insA	2
	c.4165_4166delAG	2		c.9435_9436delGT	2
	c.4956G>A	2		c.1296_1297delGA	1
	c.505C>T	2		c.1773_1776delTTAT	1
	c.5074+1G>T	2		c.1796_1800delCTTAT	1
	c.1016delA	1		c.1813_1814insA	1
	c.115T>C	1		c.3978_3979insTGCT	1
	c.1204delG	1		c.3G>A	1
	c.1916T>A	1		c.475+1G>T	1
	c.2073dupA	1		c.5238_5239insT	1
	c.2263G>T	2		c.5722_5723delCT	1
	c.2762delA	1		c.5763_5764insT	1
	c.315T>A	1		c.5763dupT	1
	c.3226delA	1		c.604_613del10	1
	c.3239T>A	1		c.6275_6276delTT	1
	c.3331C>T	1		c.6444_6445insT	1
	c.3607C>T	1		c.6444dupT	1
	c.3770_3771delAG	1		c.6447_6448dupTA	1
	c.4158_4162delCTCTC	1		c.6591_6592delTG	1
	c.4243delG	1		c.6754_6755dupT	1
	c.4539C>T	1		c.7151_7152delAA	1
	c.4689C>G	1		c.771_775delTCAAA	1
	c.4868C>G	1		c.8363G>A	1
	c.5075-2A>G	1		c.8535_8538delAGAG	1
	c.5152+1G>A	1		c.9403_9403delC	1
	c.5152+2dupT	1		c.994dupA	1
	c.5673delA	1			
	**CNVs**				
	exon 5–14del	10			
	exon 1–17del	4			
	exon 13dup	1			
	exon 13–19del	1			
	exon 18–22del	1			
	exon 21–22del	1			
	exon 21–24del	1			

No. – number of carriers; CNVs – copy number variants. *Note:* Mutation (at coding sequence) were annotated according to the reference sequence NM_007294 (for *BRCA1*) and NM_000059 (for *BRCA2*).

Table S2. Clinicopathological characteristics of all *BRCA1*/*BRCA2* mutation carriers (together and separately) and non-carriers of mutations in cancer-susceptibility genes. (*N* = denotes for individuals with known variables).

	***BRCA1*/2 Carriers (*N* = 234)**			***BRCA1* Carriers (*N* = 183)**			***BRCA2* Carriers (*N* = 51)**			**Non-Carriers (*N* = 899)**		
	N	**% ***	***p***	***N***	**% ***	***p***	***N***	**% ***	***p***	***N***	**% ***	***p***
Median age at diagnosis		(*N* = 234)			(*N* = 183)			(*N* = 51)			(*N* = 899)	
year (25–75% percentile)	37.7 (32.9–44.0)		**<0.001**	37.3 (32.8–49.5)		**<0.001**	39.7 (34.0–50.1)		0.776	40.9 (33.8–49.5)		Ref.
Age diagnosis categories		(*N* = 234)			(*N* = 183)			(*N* = 51)			(*N* = 899)	
< 35 y	86	(36.8)	**<0.001**	71	(38.8)	**<0.001**	15	(29.4)	0.718	276	(30.7)	Ref.
35–44 y	96	(41.0)		78	(42.6)		18	(35.3)		270	(30.0)	
≥ 45 y	52	(22.2)		34	(18.6)		18	(35.3)		353	(39.3)	
Menopausal status		(*N* = 234)			(*N* = 183)			(*N* = 51)			(*N* = 889)	
pre	207	(88.5)	**0.001**	166	(90.7)	**<0.001**	41	(80.4)	0.838	712	(79.2)	Ref.
post	27	(11.5)		17	(9.3)		10	(19.6)		187	(20.8)	
Primary tumor (T)		(*N* = 227)			(*N* = 180)			(*N* = 47)			(*N* = 867)	
Tis	1	(0.4)	**<0.001**	1	(0.6)	**<0.001**	0	(0.0)	0.156	45	(5.2)	Ref.
T1 (<2 cm)	78	(34.4)		57	(31.7)		21	(44.7)		417	(48.1)	
T2 (2–5 cm)	110	(48.5)		94	(52.2)		16	(34.0)		303	(34.9)	
T3 (>5 cm)	26	(11.5)		19	(10.6)		7	(14.9)		81	(9.3)	
T4	12	(5.3)		9	(5.0)		3	(6.4)		21	(2.4)	
Regional lymphatic node (N)		(*N* = 227)			(*N* = 180)			(*N* = 47)			(*N* = 867)	
N0	121	(53.3)	0.088	101	(56.1)	0.120	20	(42.6)	0.068	526	(60.7)	Ref.
N1	92	(40.5)		67	(37.2)		25	(53.2)		301	(34.7)	
N2	9	(4.0)		7	(3.9)		2	(4.3)		33	(3.8)	
N3	5	(2.2)		5	(2.8)		0	(0.0)		7	(0.8)	
Tumor stage		(*N* = 228)			(*N* = 180)			(*N* = 48)			(*N* = 889)	
0 (TisN0)	1	(0.4)	**<0.001**	1	(0.6)	**<0.001**	0	(0.0)	**0.024**	44	(4.9)	Ref.
I (T1N0–1mi)	58	(25.4)		45	(25.0)		13	(25.5)		334	(37.6)	
II (T2–3N0, T1–2N1)	123	(53.9)		101	(56.1)		22	(43.1)		389	(43.8)	
III (T3N1, TXN2–3, T4NX)	45	(19.7)		33	(18.3)		12	(23.5)		99	(11.1)	
IV (M1)	1	(0.4)		0	(0.0)		1	(2.0)		23	(2.6)	
Tumor morphology		(*N* = 232)			(*N* = 182)			(*N* = 50)			(*N* = 891)	
ductal	195	(84.1)	**0.012**	151	(83.0)	**0.001**	44	(88.0)	0.390	738	(82.8)	Ref.

lobular	6	(2.6)		3	(1.6)		3	(6.0)		70	(7.9)	
medullar	18	(7.8)		18	(9.9)		0	(0.0)		43	(4.8)	
other	13	(5.6)		10	(5.5)		3	(6.0)		40	(4.5)	
Tumor grade		(*N* = 216)			(*N* = 173)			(*N* = 43)			(*N* = 832)	
low (1)	8	(3.7)	**<0.001**	5	(2.9)	**<0.001**	3	(7.0)	0.437	115	(13.8)	Ref.
intermediate (2)	70	(32.4)		49	(28.3)		21	(48.8)		383	(46.0)	
high (3)	138	(63.9)		119	(68.8)		19	(44.2)		334	(40.1)	
ER status		(*N* = 213)			(*N* = 169)			(*N* = 44)			(*N* = 827)	
positive	72	(33.8)	**<0.001**	45	(26.6)	**<0.001**	27	(61.4)	0.519	546	(66.0)	Ref.
PR status		(*N* = 207)			(*N* = 164)			(*N* = 43)			(*N* = 799)	
positive	69	(33.3)	**<0.001**	39	(23.8)	**<0.001**	30	(69.8)	0.419	502	(62.8)	Ref.
HER-2 status		(*N* = 193)			(*N* = 152)			(*N* = 41)			(*N* = 732)	
positive	13	(6.7)	**<0.001**	10	(6.6)	**<0.001**	3	(7.3)	**0.012**	556	(24.0)	Ref.
TNBC		(*N* = 193)			(*N* = 152)			(*N* = 41)			(*N* = 729)	
yes	115	(59.6)	**<0.001**	105	(69.1)	**<0.001**	10	(24.4)	0.547	145	(19.9)	Ref.
Surgery – primary tumor		(*N* = 231)			(*N* = 182)			(*N* = 49)			(*N* = 876)	
mastectomy	122	(52.8)	0.484	94	(51.6)	0.727	28	(57.1)	0.346	440	(50.2)	Ref.
breast-conserving surgery	109	(47.2)		88	(48.4)		21	(42.9)		436	(49.8)	
Surgery – lymphatic nodes		(*N* = 227)			(*N* = 181)			(*N* = 46)			(*N* = 859)	
axillary dissection	184	(81.1)	0.054	146	(80.7)	0.105	38	(82.6)	0.294	643	(74.9)	Ref.
sentinel node biopsy	43	(18.9)		35	(19.3)		8	(17.4)		216	(25.1)	
Radiotherapy		(*N* = 229)			(*N* = 181)			(*N* = 48)			(*N* = 869)	
yes	157	(68.6)	0.475	128	(70.7)	0.225	29	(60.4)	0.423	574	(66.1)	Ref.
Chemotherapy type		(*N* = 232)			(*N* = 182)			(*N* = 50)			(*N* = 873)	
Antra + Tax	122	(52.6)	**<0.001**	97	(53.3)	**<0.001**	25	(50.0)	0.214	311	(35.6)	Ref.
Antra	61	(26.3)		49	(26.9)		12	(24.0)		243	(27.8)	
Other	21	(9.1)		17	(9.3)		4	(8.0)		85	(9.7)	
No chemotherapy	28	(12.1)		19	(10.4)		9	(18.0)		234	(26.8)	
Endocrine therapy **		(*N* = 65)			(*N* = 38)			(*N* = 27)			(*N* = 490)	
TAM monotherapy	26	(40.0)	0.961	18	(47.4)	0.886	8	(29.6)	0.655	203	(41.4)	Ref.
AI monotherapy	8	(12.3)		4	(10.5)		4	(14.8)		66	(13.5)	
LHRH analogues + TAM	29	(44.6)		15	(39.5)		14	(51.9)		210	(42.9)	
LHRH analogues + AI	2	(3.1)		1	(2.6)		1	(3.7)		11	(2.2)	
Event during follow-up ***		(*N* = 233)			(*N* = 183)			(*N* = 51)			(*N* = 876)	

loco-regional recurrence	14	(6.0)	0.169	13	(7.1)	0.457	1	(2.0)	0.093	77	(8.8)	Ref.
distant metastasis	47	(20.2)	**0.002**	28	(15.3)	0.289	19	(38.0)	**0.027**	110	(12.6)	Ref.
second breast cancer	43	(18.5)	**<0.001**	34	(18.6)	**<0.001**	9	(18.0)	**0.026**	76	(8.7)	Ref.
second tumors	12	(5.2)	0.753	10	(5.5)	0.805	2	(4.0)	0.570	52	(5.9)	Ref.
Median of follow-up (years)	(*N* = 234)			(*N* = 183)			(*N* = 51)			(*N* = 899)		
median (25–75% percentil)	10.0 (6.3-16.0)		0.235	9.6 (6.14–15.44)		0.692	11.2 (7.7–17.3)		**0.048**	9.8 (5.9–15.8)		Ref.
Breast cancer-related death	(*N* = 234)			(*N* = 183)			(*N* = 51)			(*N* = 899)		
yes	32	(13.7)	0.507	22	(12.0)	0.429	13	(25.5)	**0.028**	128	(14.2)	Ref.

* % = percentage of known; ** *N* = number of patients with ER-positive BC; *** patient could be counted in more than one event; pre – premenopausal; post – postmenopausal; TNBC – triple-negative BC; Antra – anthracyclines; Tax – taxanes; TAM – tamoxifen; AI – aromatase inhibitor; LHRH - luteinizing hormone-releasing hormone; Ref. – reference.

Table S3. Analysis of 10-year DFS and DSS using the Mantel-Haenszel test comparing variables within subgroups of *BRCA1/2* mutation carriers and non-carriers, respectively.

Variable	Category	**Disease Free Survival (DFS) Analysis**												**Disease Specific Survival (DSS) Analysis**											
		***BRCA1*/2 Carriers**						**Non-carriers**						***BRCA1*/2 Carriers**						**Non-Carriers**					
		**Pts No.**	**Ev No.**	**Ev %**	**HR**	**95% CI**	***p***	**Pts No.**	**Ev No.**	**Ev %**	**HR**	**95% CI**	***p***	**Pts No.**	**Ev No.**	**%**	**HR**	**95% CI**	***p***	**Pts No.**	**Ev No.**	**%**	**HR**	**95% CI**	***p***
**All Pts**		191	46	24.1	-	-	-	680	128	18.8	-	-	-	191	28	14.7	-	-	-	680	64	9.4	-	-	-
**Age at diagnosis**	<35	78	18	23.1	0.98	0.55–1.78	0.960	213	60	28.2	**2.21**	**1.51–3.22**	**<0.001**	78	12	15.4	1.16	0.54–2.48	0.703	213	32	15.0	**2.29**	**1.35–3.87**	**0.002**
	≥35	113	28	24.8	Ref.			467	68	14.6	Ref.			113	16	14.2	Ref.			467	32	6.9	Ref.		
	<45	150	34	22.7	0.84	0.42–1.67	0.612	422	100	23.7	**2.17**	**1.53–3.09**	**<0.001**	150	21	14.0	0.88	0.36–2.13	0.779	422	49	11.6	**1.98**	**1.20–3.26**	**0.008**
	≥45	41	12	29.3	Ref.			258	28	10.9	Ref.			41	7	17.1	Ref.			258	15	5.8	Ref.		
**Menopausal status**	pre	168	39	23.2	0.80	0.34–1.90	0.611	532	119	22.4	**1.71**	**1.15–2.56**	**0.009**	168	23	13.7	0.62	0.20–1.90	0.406	532	55	10.3	1.53	0.88–2.64	0.129
	post	23	7	30.4	Ref.			148	18	12.2	Ref.			23	5	21.7	Ref.			148	9	6.1	Ref.		
**Tumor size**	T1	71	11	15.5	Ref.			355	45	12.7	Ref.			71	4	5.6	Ref.			355	18	5.1	Ref.		
	T2	84	20	23.8	1.63	0.80–2.29	0.176	244	52	21.3	**1.94**	**1.28–2.92**	**0.002**	84	12	14.3	2.53	0.95–6.76	0.063	244	29	11.9	**2.76**	**1.53–4.98**	**0.001**
	T3	24	9	37.5	**3.45**	**1.22–9.77**	**0.020**	62	23	37.1	**7.25**	**3.52–14.93**	**<0.001**	24	7	29.2	**7.91**	**2.02–31.0**	**0.003**	62	13	21.0	**12.4**	**4.38–35.2**	**<0.001**
	T4	12	6	50.0	**13.18**	**2.83–61.48**	**0.001**	19	8	42.1	**19.71**	**4.91–79.13**	**<0.001**	12	5	41.7	**94.9**	**11.8–766**	**<0.001**	19	4	21.1	**31.7**	**3.96–253**	**0.001**
**Nodal status**	N0	100	17	17.0	Ref.			387	51	13.2	Ref.			100	11	11.0	Ref.			387	19	4.9	Ref.		
	N1	78	25	32.1	**2.35**	**1.27–4.38**	**0.007**	256	64	25.0	**2.17**	**1.49–3.16**	**<0.001**	78	15	19.2	2.12	0.96–4.65	0.062	256	38	14.8	**3.40**	**2.00–5.80**	**<0.001**
	N2	8	2	25.0	1.62	0.29–9.12	0.587	30	9	30.0	**5.19**	**1.77–15.21**	**0.003**	8	1	12.5	1.26	0.13–11.8	0.842	30	4	13.3	**6.09**	**1.16–32.1**	**0.033**
	N3	5	2	40.0	5.56	0.56–55.54	0.144	7	4	57.1	**141.7**	**12.93–1553**	**<0.001**	5	1	20.0	2.94	0.17–51.1	0.459	7	3	42.9	-	-	-
**Tumor stage**	I	52	7	13.5	Ref.			282	28	9.9	Ref.			52	2	3.8	Ref.			282	8	2.8	Ref.		
	II	97	23	23.7	1.78	0.85–3.74	0.128	310	65	21.0	**2.26**	**1.50–3.39**	**<0.001**	97	15	15.5	**2.86**	**1.06–7.71**	**0.038 38**	310	37	11.9	**3.61**	**2.01–6.48**	**<0.001**
	III	42	16	38.1	**3.25**	**1.41–7.46**	**0.006**	88	35	39.8	**10.35**	**5.53–19.38**	**<0.001**	42	11	26.2	**5.96**	**1.97–19.0**	**0.002**	88	19	21.6	**19.6**	**7.74–49.4**	**<0.001**
**Tumor grade**	1	7	2	28.6	Ref.			91	6	6.6	Ref.			7	1	14.3	Ref.			91	1	1.1	Ref.		
	2	59	16	27.1	1.01	0.23–4.38	0.986	311	59	19.0	**2.21**	**1.24–3.92**	**0.007**	59	9	15.3	1.20	0.18–8.14	0.856	311	21	6.8	2.75	1.00–7.57	0.051
	3	125	28	22.4	0.89	0.20–4.01	0.880	278	63	22.7	**2.55**	**1.50–4.33**	**0.001**	125	18	14.4	1.16	0.18–7.59	0.874	278	42	15.1	**3.44**	**1.74–6.79**	**<0.001**
**ER status**	pos	60	23	38.3	**2.33**	**1.25–4.33**	**0.008**	445	74	16.6	**0.66**	**0.46–0.95**	**0.027**	60	13	21.7	1.82	0.83–4.00	0.136	445	28	6.3	**0.37**	**0.22–0.62**	**<0.001**
	neg	131	23	17.6	Ref.			235	54	23.0	Ref.			131	15	11.5	Ref.			235	36	15.3	Ref.		
**PR status**	pos	62	22	35.5	**2.01**	**1.09–3.73**	**0.026**	428	76	17.8	0.79	0.55–1.13	0.200	62	10	16.1	1.07	0.49–2.34	0.861	428	30	7.0	**0.46**	**0.27–0.77**	**0.003**
	neg	129	24	18.6	Ref.			252	52	20.6	Ref.			129	18	14.0	Ref.			252	34	13.5	Ref.		
**HER-2 status**	pos	13	4	30.8	1.57	0.47–5.25	0.464	164	34	20.7	1.21	0.86–2.08	0.369	13	2	15.4	1.18	0.25–5.50	0.832	164	19	11.6	1.46	0.81–2.61	0.205
	neg	178	42	23.6	Ref.			516	94	18.2	Ref.			178	26	14.6	Ref.			516	45	8.7	Ref.		
**TNBC**	yes	114	20	17.5	**0.50**	**0.28–0.90**	**0.021**	138	30	21.7	1.34	0.86–2.08	0.197	114	14	12.3	0.70	0.33–1.48	0.350	138	21	15.2	**2.40**	**1.29–4.46**	**0.006**
	no	77	26	33.8	Ref.			542	98	18.1	Ref.			77	14	18.2	Ref.			542	43	7.9	Ref.		

Pts No. – number of patients; Ev No. – number of events; Ev % - percentage of events; HR – hazard ratio; 95% CI – 95% confidential interval; pre – premenopausal; post – postmenopausal; pos – positive; neg – negative; Ref. – reference.

Table S4. Analysis of 10-year DFS and DSS using a univariate Cox regression model comparing variables within subgroups of *BRCA1/2* mutation carriers and non-carriers.

Variable	Category	Disease Free Survival (DFS) Analysis						Disease Specific Survival (DSS) Analysis					
		BRCA1/2 Carriers			Non-Carriers			*BRCA1/2* Carriers			Non-Carriers		
		HR	95% CI	*p*	HR	95% CI	*p*	HR	95% CI	*p*	HR	95% CI	*p*
**Age at diagnosis**	continuous	1.01	0.98–1.04	0.564	**0.96**	**0.95–0.98**	**<0.001**	1.00	0.96–1.04	0.975	**0.96**	**0.93–0.98**	**0.002**
	<35 vs ≥35 (Ref.)	0.98	0.55–1.78	0.960	**2.04**	**1.44–2.89**	**<0.001**	1.16	0.55–2.45	0.703	**2.13**	**1.30–3.47**	**0.003**
	<45 vs ≥45 (Ref.)	0.84	0.44–1.63	0.612	**2.44**	**1.60–3.71**	**<0.001**	0.88	0.38–2.08	0.780	**2.16**	**1.21–3.84**	**0.009**
**menopausal status**	pre vs post (Ref.)	0.81	0.36–1.81	0.611	**1.92**	**1.15–3.20**	**0.012**	0.67	0.25–1.75	0.409	1.74	0.86–3.53	0.122
**Tumor size**	T2–4 vs T1 (Ref.)	**2.09**	**1.06–4.12**	**0.033**	**2.30**	**1.60–3.30**	**<0.001**	**3.92**	**1.36–11.3**	**0.011**	**3.18**	**1.84–5.49**	**<0.001**
	T3–4 vs T1–2 (Ref.)	**2.43**	**1.31–4.51**	**0.005**	**2.82**	**1.88–4.23**	**<0.001**	**3.55**	**1.68–7.51**	**0.001**	**2.97**	**1.70–5.17**	**<0.001**
	T4 vs T1–3 (Ref.)	**3.00**	**1.27–7.08**	**0.012**	**2.76**	**1.35–5.65**	**0.005**	**4.34**	**1.64–11.4**	**0.003**	**2.73**	**0.99–7.50**	**0.052**
**Nodal status**	N1–3 vs N0 (Ref.)	**2.23**	**1.23–4.07**	**0.009**	**2.23**	**1.57–3.18**	**<0.001**	1.97	0.92–4.21	0.080	**3.43**	**2.01–5.87**	**<0.001**
	N2–3 vs N0–1 (Ref.)	1.30	0.47–3.64	0.613	**2.41**	**1.36–4.27**	**0.003**	1.10	0.26–4.63	0.898	**2.44**	**1.11–5.35**	**0.026**
	N3 vs N0–2 (Ref.)	1.90	0.46–7.86	0.373	**4.00**	**1.48–10.9**	**0.006**	1.57	0.21–11.6	0.658	**6.76**	**2.11–21.6**	**0.001**
**Tumor stage**	II–III vs I (Ref.)	**2.30**	**1.03–5.13**	**0.043**	**2.90**	**1.91–4.42**	**<0.001**	**5.26**	**1.25–22.2**	**0.024**	**5.55**	**2.65–11.7**	**<0.001**
	III vs I–II (Ref.)	**2.16**	**1.18–3.97**	**0.013**	**3.12**	**2.12–4.61**	**<0.001**	**2.58**	**1.21–5.50**	**0.015**	**3.22**	**1.88–5.51**	**<0.001**
**Tumor grade**	2–3 vs 1 (Ref.)	0.94	0.23–3.88	0.933	**3.44**	**1.52–7.82**	**0.003**	1.20	0.16–8.81	0.860	**9.97**	**1.38–71.9**	**0.023**
	3 vs 1–2 (Ref.)	0.90	0.50–1.63	0.728	**1.57**	**1.11–2.22**	**0.011**	1.07	0.49–2.31	0.870	**3.10**	**1.85–5.20**	**<0.001**
**ER status**	pos vs neg (Ref.)	**2.15**	**1.21–3.84**	**0.009**	**0.67**	**0.47–0.96**	**0.028**	1.75	0.83–3.67	0.141	**0.40**	**0.24–0.65**	**<0.001**
**PR status**	pos vs neg (Ref.)	**1.91**	**1.07–3.40**	**0.029**	0.79	0.56–1.13	0.201	1.07	0.49–2.32	0.861	**0.48**	**0.29–0.79**	**0.004**
**HER–2 status**	pos vs neg (Ref.)	1.46	0.52–4.09	0.467	1.20	0.81–1.77	0.370	1.17	0.28–4.93	0.832	1.41	0.83–2.42	0.206
**TNBC**	yes vs no (Ref.)	**0.51**	**0.28–0.91**	**0.024**	1.31	0.87–1.97	0.198	0.70	0.34–1.48	0.352	**2.05**	**1.22–3.45**	**0.007**

Pts No. – number of patients; Ev No. – number of events; OR – odds ratio; 95% CI – 95% confidential interval; pre – premenopausal; post – postmenopausal; pos – positive; neg – negative; Ref. – reference.

Table S5. Analysis of 10-year DFS and DSS using multivariable Cox proportional-hazard models adjusted for age, menopausal status, stage, tumor grade and ER status.

	***BRCA1/2* Carriers**						**Non-Carriers**					
	**DFS**			**DSS**			**DFS**			**DSS**		
	**HR**	**95%CI**	***p*-Value**	**HR**	**95%CI**	***p*-Value**	**HR**	**95%CI**	***p*-Value**	**HR**	**95%CI**	***p*-Value**
**model A**												
Age (continuous)	1.01	0.97–1.06	0.552	0.99	0.94–1.04	0.661	**0.96**	**0.93–0.98**	**0.001**	**0.95**	**0.92–0.99**	**0.014**
Menopausal status (post vs pre)	1.04	0.32–3.38	0.948	0.41	0.10–1.77	0.234	0.60	0.27–1.32	0.205	0.44	0.14–1.34	0.148
Tumor stage (II–III vs I)	**2.38**	**1.04–5.48**	**0.041**	**5.63**	**1.30–24.4**	**0.021**	**2.54**	**1.66–3.89**	**<0.001**	**4.56**	**2.16–9.63**	**<0.001**
Tumor grade (3 vs 1–2)	1.20	0.64–2.27	0.571	1.36	0.59–3.17	0.470	1.10	0.75–1.63	0.629	**1.92**	**1.09–3.38**	**0.025**
ER–status (pos vs neg)	**2.19**	**1.17–4.09**	**0.014**	1.70	0.76–3.82	0.197	0.76	0.51–1.12	0.161	**0.57**	**0.33–0.98**	**0.041**
**model B**												
Age (<35 vs ≥35)	0.92	0.49–1.74	0.796	1.18	0.52–2.70	0.692	**1.70**	**1.17–2.48**	**0.005**	**1.67**	**0.98–2.87**	**0.060**
Menopausal status (post vs pre)	0.84	0.34–2.06	0.707	0.48	0.16–1.44	0.191	1.24	0.71–2.16	0.442	1.01	0.46–2.18	0.987
Tumor stage (II–III vs I)	**2.33**	**1.02–5.35**	**0.045**	**5.75**	**1.34–24.8**	**0.019**	**2.61**	**1.71–4.01**	**<0.001**	**4.73**	**2.24–9.99**	**<0.001**
Tumor grade (3 vs 1–2)	1.19	0.63–2.25	0.593	1.37	0.59–3.17	0.468	1.11	0.75–1.64	0.594	**1.91**	**1.08–3.37**	**0.025**
ER–status (pos vs neg)	**2.19**	**1.17–4.11**	**0.015**	1.70	0.76–3.82	0.200	0.76	0.51–1.12	0.169	**0.57**	**0.33–0.97**	**0.039**
**model C**												
Age (<45 vs ≥45)	0.92	0.38–2.23	0.847	1.14	0.34–3.83	0.830	**2.41**	**1.30–4.48**	**0.005**	1.86	0.81–4.27	0.141
menopausal status (post vs pre)	0.87	0.29–2.60	0.801	0.47	0.14–1.88	0.287	0.76	0.36–1.62	0.481	0.78	0.29–2.14	0.632
Tumor stage (II–III vs I)	**2.33**	**1.02–5.32**	**0.046**	**5.76**	**1.34–24.8**	**0.019**	**2.53**	**1.65–3.88**	**0.000**	**4.57**	**2.16–9.67**	**<0.001**
Tumor grade (3 vs 1–2)	1.18	0.63–2.23	0.602	1.38	0.60–3.19	0.452	1.12	0.76–1.65	0.578	**1.93**	**1.09–3.41**	**0.023**
ER–status (pos vs neg)	**2.17**	**1.16–4.05**	**0.015**	1.73	0.77–3.88	0.181	0.74	0.50–1.10	0.137	**0.56**	**0.32–0.95**	**0.033**
**model D**												
Age (continuous)	1.01	0.96–1.05	0.758	0.98	0.92–1.03	0.426	**0.96**	**0.93–0.98**	**0.001**	**0.95**	**0.91–0.99**	**0.008**
Menopausal status (post vs pre)	1.00	0.29–3.39	0.995	0.37	0.08–1.75	0.209	0.65	0.30–1.41	0.276	0.47	0.15–1.42	0.181
Tumor stage (III vs I–II)	**1.93**	**1.01–3.67**	**0.047**	**2.50**	**1.11–5.63**	**0.026**	**2.99**	**2.00–4.47**	**0.000**	**2.78**	**1.60–4.85**	**<0.001**
Tumor grade (3 vs 1–2)	1.10	0.57–2.11	0.775	1.15	0.48–2.72	0.755	1.02	0.69–1.52	0.918	**1.81**	**1.01–3.24**	**0.047**
ER–status (pos vs neg)	**1.97**	**1.02–3.78**	**0.043**	1.48	0.64–3.45	0.361	**0.66**	**0.45–0.98**	**0.039**	**0.50**	**0.29–0.87**	**0.014**
**model E**												
Age (<35 vs ≥35)	1.03	0.54–1.94	0.937	1.32	0.58–3.03	0.505	**1.63**	**1.12–2.39**	**0.011**	1.59	0.92–2.73	0.094
Menopausal status (post vs pre)	0.86	0.35–2.11	0.735	0.52	0.17–1.57	0.245	1.43	0.82–2.48	0.203	1.18	0.55–2.55	0.673
Tumor stage (III vs I–II)	**1.93**	**1.01–3.68**	**0.047**	**2.56**	**1.14–5.75**	**0.023**	**2.90**	**1.94–4.34**	**0.000**	**2.70**	**1.55–4.71**	**<0.001**
Tumor grade (3 vs 1–2)	1.08	0.56–2.08	0.811	1.15	0.49–2.72	0.752	1.06	0.71–1.57	0.773	**1.88**	**1.05–3.37**	**0.033**
ER–status (pos vs neg)	**1.96**	**1.02–3.78**	**0.044**	1.46	0.63–3.41	0.378	0.69	0.47–1.02	0.061	**0.52**	**0.30–0.90**	**0.019**
**model F**												
Age (<45 vs ≥45)	0.90	0.36–2.24	0.828	1.19	0.32–4.37	0.795	**2.73**	**1.47–5.09**	**0.002**	2.21	0.96–5.09	0.064
Menopausal status (post vs pre)	0.94	0.31–2.87	0.913	0.51	0.12–2.28	0.381	0.78	0.36–1.65	0.510	0.77	0.28–2.15	0.624
Tumor stage (III vs I–II)	**1.94**	**1.01–3.69**	**0.045**	**2.50**	**1.11–5.65**	**0.027**	**3.10**	**2.07–4.63**	**0.000**	**2.82**	**1.62–4.90**	**<0.001**
Tumor grade (3 vs 1–2)	1.09	0.57–2.08	0.794	1.19	0.51–2.79	0.691	1.06	0.71–1.57	0.782	**1.90**	**1.06–3.40**	**0.031**
ER–status (pos vs neg)	**1.96**	**1.02–3.77**	**0.045**	1.50	0.64–3.49	0.352	**0.66**	**0.45–0.98**	**0.038**	**0.51**	**0.29–0.87**	**0.015**

Please add footnote here to explain the bold text in the table.

Table S6. The indication criteria for genetic testing of hereditary BC/OC predisposition in the Czech Republic.

**Familial Indication Criteria (IC):**
• ≥ 3 first and/or second-degree relatives with BC (including the proband)
• 2 first and/or second-degree relatives with BC (1 diagnosed at ≤ 50 years, or both diagnosed at ≤ 60 years, or one with male BC; including the proband)
• BC and OC in first and/or second-degree relatives (including the proband), or BC ≤ 50 years and PaC (including the proband)
• ≥ 2 first and/or second-degree relatives with OC and PaC (including the proband)
• ≥ 2 first and/or second-degree relatives with OC (including the proband)
**Personal (Individual) Indication Criteria:**
• BC diagnosed at ≤ 45 years (or at ≤ 50 years, when family history is unknown or limited)
• BC duplicity (first cancer diagnosed at ≤ 50 years or both cancers at ≤ 60 years)
• "Triple negative" BC diagnosed at ≤ 60 years
• Medullary BC
• Male BC
• Duplicity of BC and OC
• Duplicity of BC and PaC
• OC at any age

BC – breast cancer; OC – ovarian cancer (including the fallopian tube and primary peritoneal cancer); PaC – pancreatic cancer; close blood relatives include first-, second-, and/or third-degree relatives from the same family side. *Note:* The Czech national guidelines are based on NCC*N* guidelines (www.nccn.org) and since 2003 updated by the consensus of the expert panel constituted from the members of Czech Society for Oncology (www.linkos.cz) and Society of Medical Genetics and Genomics (www.slg.cz) [1–5]. The guidelines used for the enrollment of high-risk patients before 2003 were described by Pohlreich et al. [6,7].

Reference

1. Bartonkova, H.; Foretová, L.; Helmichová, E.; Kalábová, R.; Kleibl, Z.; Konopásek, B.; Krutílková, V.; Machackova, E.; Novotny, J.; Petráková, K. Recommendations for care of patients with breast and ovaria*N* cancer and healthy individuals with germline mutations in BRCA1 or BRCA2. *Klin. Onkol.* **2003**, *16*, 28–34.
2. Plevová, P.; Novotný, J.; Petráková, K.; Palácová, M.; Kalábová, R.; Schneiderová, M.; Foretová, L.; Hereditary Breast and Ovarian Cancer Syndrome. *Klin. Onkol.* **2009**, *22*, 8–11.
3. Pohlreich, P.; Kleibl, Z.; Kleiblová, P.; Janatová, M.; Soukupová, J.; Macháčková, E.; Házová, J.; Vašíčková, P.; Sťahlová, H.E.; Navrátilová, M.; et al. The Clinical Importance of a Genetic Analysis of Moderate-Risk Cancer Susceptibility Genes in Breast and Other Cancer Patients from the Czech Republic *Klin. Onkol.* **2012**, *25 Suppl 1*, S59–S66.
4. Janatova, M.; Borecka, M.; Soukupova, J.; Kleiblova, P.; Stribrna, J.; Vocka, M.; Zemánková, P.; Panczak, A.; Veselá, K.; Souček, P.; et al. PALB2 as Another Candidate Gene for Genetic Testing in Patients with Hereditary Breast Cancer in Czech Republic. *Klin. Onkol.* **2016**, *29 Suppl 1*, 31–34.
5. Foretova, L.; Machackova, E.; Palacova, M.; Navratilova, M.; Svoboda, M.; Petrakova, K. Recommended Extension of Indication Criteria for Genetic Testing of BRCA1 and BRCA2 Mutations in Hereditary Breast and Ovarian Cancer Syndrome. *Klin. Onkol.* **2016**, *29 Suppl 1*, S9–S13.
6. Pohlreich, P.; Stribrna, J.; Kleibl, Z.; Zikan, M.; Kalbacova, R.; Petruzelka, L.; Konopasek, B. Mutations of the BRCA1 gene in hereditary breast and ovarian cancer in the Czech Republic. *Med. Princ. Pract.* **2003**, *12*, 23–29.
7. Pohlreich, P.; Stribrna, J.; Zikan, M.; Kleibl, Z.; Matous, B.; Novotny, J. Analysis of BRCA1 and BRCA2 mutations in high-risk patients from the Prague-area. *Eur. J. Cancer Suppl.* **2005**, *3*, 89.

© 2019 by the authors. Submitted for possible ope*N* access publicatio*N* under the terms and conditions of the Creative Commons Attributio*N* (CC BY) license (http://creativecommons.org/licenses/by/4.0/).
